# Structures of Get3d reveal a distinct architecture associated with the emergence of photosynthesis

**DOI:** 10.1016/j.jbc.2023.104752

**Published:** 2023-04-24

**Authors:** Alexandra N. Barlow, M.S. Manu, Shyam M. Saladi, Paul T. Tarr, Yashpal Yadav, Aye M.M. Thinn, Yun Zhu, Arthur D. Laganowsky, William M. Clemons, Sureshkumar Ramasamy

**Affiliations:** 1 Division of Chemistry and Chemical Engineering, California Institute of Technology, Pasadena, California, USA; 2 Division of Biochemical Sciences, CSIR-National Chemical Laboratory, Pune, India; 3 Howard Hughes Medical Institute and Division of Biology and Biological Engineering, California Institute of Technology, Pasadena, California, USA; 4 Department of Chemistry, Texas A&M University, College Station, Texas, USA

**Keywords:** cyanobacteria, GET pathway, Get3, protein targeting, protein structure, plant, NTPase, structural biology, tail-anchored protein

## Abstract

Homologs of the protein Get3 have been identified in all domains yet remain to be fully characterized. In the eukaryotic cytoplasm, Get3 delivers tail-anchored (TA) integral membrane proteins, defined by a single transmembrane helix at their C terminus, to the endoplasmic reticulum. While most eukaryotes have a single Get3 gene, plants are notable for having multiple Get3 paralogs. Get3d is conserved across land plants and photosynthetic bacteria and includes a distinctive C-terminal α-crystallin domain. After tracing the evolutionary origin of Get3d, we solve the *Arabidopsis thaliana* Get3d crystal structure, identify its localization to the chloroplast, and provide evidence for a role in TA protein binding. The structure is identical to that of a cyanobacterial Get3 homolog, which is further refined here. Distinct features of Get3d include an incomplete active site, a “closed” conformation in the apo-state, and a hydrophobic chamber. Both homologs have ATPase activity and are capable of binding TA proteins, supporting a potential role in TA protein targeting. Get3d is first found with the development of photosynthesis and conserved across 1.2 billion years into the chloroplasts of higher plants across the evolution of photosynthesis suggesting a role in the homeostasis of photosynthetic machinery.

A central problem for eukaryotes and photosynthetic microbes is the presence of multiple membranes that contain specific subsets of integral membrane proteins (IMPs). The correct targeting and insertion of IMPs to distinct and specific locations is necessary to maintain cellular homeostasis (1). Most IMPs span the lipid bilayer with hydrophobic α-helical transmembrane domains (TMDs) that present a challenge during biogenesis, as they must be shielded from the aqueous environment prior to insertion to avoid aggregation. For IMPs, a signal, often the first TMD, encodes the destination, and the location of the signal in the protein sequence dictates cotranslational *versus* post-translational targeting.

A subset of IMPs, termed tail-anchored (TA) proteins, are defined by a single TMD within ∼30 residues of the C terminus (2), which serves as their targeting signal. Because of the position of their TMDs in the sequence, TA proteins must be fully synthesized and released by the ribosome prior to targeting (3). TA proteins are targeted in a post-translational chaperone-assisted mechanism primarily by the Guided Entry of TA proteins (GET) pathway. A central player in the GET pathway is Get3, which captures the TA signal in the cytosol and delivers the protein to a membrane insertion complex at the endoplasmic reticulum (ER), dependent on ATP hydrolysis (4, 5, 6). Get3 has been shown to have a conserved mechanism across eukaryotes (5, 6). Many TA proteins play essential roles, for example, vesicle trafficking, protein localization, and regulation of apoptosis, and are targeted to various membranes (7, 8).

Organisms that generate energy *via* photosynthesis require additional cellular compartments and membranes, which necessitates added complexity for the targeting of TA proteins to these membranes (9). Examples are chloroplasts and other plastids, multimembrane organelles, derived from a single endosymbiotic event in which a cyanobacterial ancestor was incorporated into an early eukaryote, around 1.2 billion years ago (10). Given this, it is unsurprising that protein targeting in chloroplasts has features conserved from protein targeting in bacteria. While much is known about membrane protein targeting in chloroplasts (11, 12), it is unclear if a distinct mechanism exists for targeting of TA proteins to either the thylakoid or inner envelope membrane of chloroplasts. Interesting chloroplastic TA protein examples are the two SecE paralogs of the distinct Sec translocons present on either the inner envelope (SECE2) or thylakoid (SECE1) membranes where the respective targeting was found to be dependent solely on the TMD and flanking C-terminal residues (13). Recently, the Get3 paralog *At*Get3b has been implicated in targeting SECE1 to the thylakoid membrane (14); however, further studies will be necessary to probe the role of *At*Get3b *in vivo*. While the protein factors and specific targeting mechanisms are not yet known, sorting mechanisms are required that distinguish the characteristics of the TA proteins (14, 15).

Sequence analysis revealed that the Get3/ArsA fold family includes homologs from all three domains of life including one identified by Pfam in 2006 (16) and discussed first by Chartron *et al.* in 2012 (17) with a distinct architecture characterized by an α-crystallin domain (αCD) at its C terminus. Unlike fungi and metazoa, which contain a single Get3 in each genome, plants and cyanobacteria have multiple Get3 genes (8, 17, 18, 19, 20). The model plant *Arabidopsis thaliana* (*A. thaliana*) contains four such genes (noted Get3a, b, c, and d). Evidence supports that *At*Get3a (UniProt ID: Q949M9) resides in the cytosol, *At*Get3b (UniProt ID: A1L4Y1) in the chloroplast stroma, and *At*Get3c (UniProt ID: Q5XF80) in the mitochondrial matrix (18, 19, 20, 21, 22). *At*Get3a has the same function as other cytoplasmic Get3 homologs in eukaryotes, targeting hydrophobic TA proteins in the cytosol to the ER membrane with knockouts of *At*Get3a resulting in distinct phenotypes (20, 23). There is conflicting evidence as to whether *At*Get3b knockouts cause a phenotypic defect (14, 20), and no phenotype has been found for knockouts of *At*Get3c (20). No experimental information is available for *At*Get3d (UniProt ID: Q6DYE4).

Here, we provide a detailed characterization of Get3d, a distinct member of the Get3 family. We demonstrate that it first evolved in photosynthetic bacteria and has been conserved in the chloroplasts of plants. We solve the atomic structure of a plant Get3d and further refine a previously deposited structure of a cyanobacterial Get3d. We identify conserved functional motifs and identify distinct features of Get3d. We then investigate these functional motifs and show that Get3d can bind TA proteins irrespective of the unique αCD at its C terminus and can hydrolyze ATP. This work provides a comprehensive characterization of a Get3 family member that has deep evolutionary roots connected to photosynthesis.

## Results

### Placing plant paralogs within the Get3/ArsA fold family

**Figure 1:**
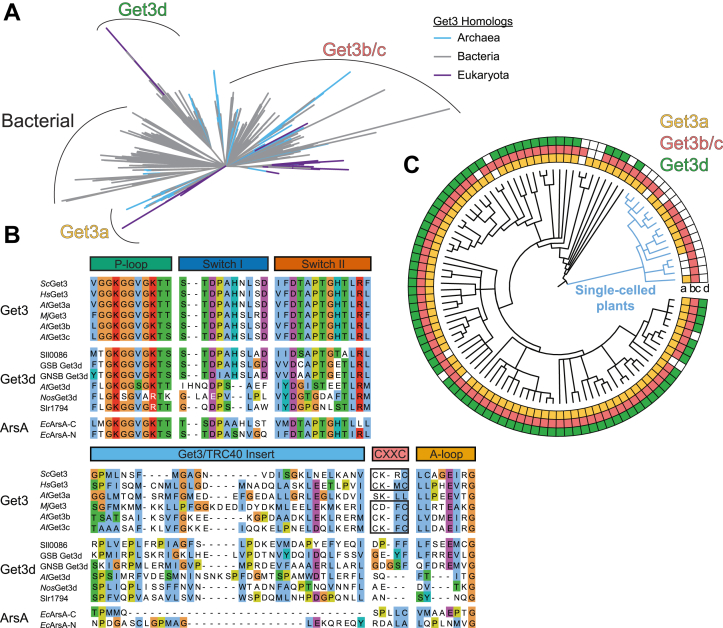
**Identification and features of Get3 homologs.***A*, a phylogenetic tree of Get3 homologs where branchpoints (*i.e.*, nodes) with less than 70% bootstrap support are collapsed. Get3d is found in a separate clade. Get3a clusters with canonical cytoplasmic Get3 proteins, including yeast Get3 (UniProt ID: Q12154). Get3b and Get3c also form a distinct clade. Superkingdom is highlighted by color Archaea (*light blue*), Bacteria (*gray*), and Eukaryota (*purple*). Inner branches are colored where all descendants are of a single taxonomic grouping. *B*, sequence alignment of important regions from selected Get3 homologs. Features as discussed in the text are labeled above the respective sequences. Residues are colored per the ClustalX color scheme (72). Species key: *At*, *Arabidopsis thaliana*; *Ec*, *Escherichia coli*; GSB, *Chlorobium* sp. (green sulfur bacteria); GNSB, *Chloroflexi* sp. (green nonsulfur bacteria); *Hs*, *Homo sapiens; Mj*, *Methanocaldococcus jannaschii*; *Nos*, *Nostoc* sp. PCC 7120; *Sc*, *Saccharomyces cerevisiae*. Slr1794 and Sll0086 are Get3d homologs from *Synechocystis* sp. PCC 6803. *C*, a cladogram of plants showing the presence of Get3 homologs by taxonomic genera. *Filled squares* show that a homolog was identified in at least one member of the genus. Clades are colored Get3a (*yellow*), Get3b/c (*red*), and Get3d (*green*). Single-celled plants are labeled and highlighted as *blue branches* in the tree. A higher resolution figure with labeled genera is provided in Fig. S3.

As Get3/ArsA homologs span the tree of life with several distinct clade lineages, we first sought to examine the evolutionary history of Get3d by performing a thorough phylogenetic reconstruction of Get3/ArsA homologs (Figs. 1*A* and S1). We identified all Get3 proteins present in UniProt using the Pfam database (16, 22) and then aligned them to a seed structural alignment of Get3 proteins based on solved 3D structures (24, 25). A phylogenetic reconstruction was then calculated using maximum likelihood with clades collapsed at a 70% bootstrap support (26).

The first clade is ArsA, a soluble ATPase involved in protecting against arsenite toxicity found primarily in bacteria (27, 28, 29, 30, 31). Next is the well-studied cytoplasmic Get3 that is present across eukaryotes and some archaea and includes *At*Get3a. Another Get3 lineage is restricted to Viridiplantae and contains both *At*Get3b and *At*Get3c. The final Get3 lineage is first found in green and purple bacteria and then cyanobacteria and across Viridiplantae and contains both *At*Get3d and the Get3d homolog from the cyanobacteria *Nostoc* sp. PCC 7120 (*Nos*Get3d) with a solved structure (Figs. 1*A* and S1).

At the sequence level, Get3d proteins contain regions that can be aligned to notable motifs of the Get3 family, including the P-loop, switch-I, switch-II, Get3 motif/TRC40-insert, and A-loop (Fig. 1*B*) (32). The P-loop is well conserved with the so-called intradimeric (or “deviant”) Walker A motif (28, 31, 33). Some Get3d proteins, such as the second homolog in *Synechocystis* sp. PCC 6803 (Sll0086) and green sulfur and nonsulfur Get3d, have well-conserved switch I, switch II, and A-loop regions. More common are homologs such as *At*Get3d and *Nos*Get3d that have degenerate catalytic residues including divergent switch I loops and missing A-loops. The hydrophobic nature of the Get3 motif/TRC40-insert of Get3 homologs is conserved across the Get3d family, unlike the related ArsA proteins (32). The signature CXXC motif that coordinates a Zn^2+^ at the Get3 dimer interface is also missing in the Get3d family (32).

To better understand the origin and distribution of Get3 proteins from plants and photosynthetic bacteria, we carried out a more extensive phylogenetic reconstruction by specifically focusing on Get3 proteins from these two groups. Plant Get3a, Get3b/Get3c, as well as Get3d homologs, each form separate clades (Fig. S2, *A*–*C*). Get3a forms a single clade with fungal and metazoan Get3 homologs found at their root (Figs. S1 and S2*A*). Get3b and Get3c trace to a single common ancestor, that is, monophyletic, and the clade cannot be split into separate b and c groups (Figs. S1 and S2*B*). Get3d forms a single clade with cyanobacteria and the green sulfur *Chloroflexi* Get3 proteins as the nearest relatives (Figs. S1 and S2*C*). Get3a and Get3b/c clades trace to a more recent common ancestor before their common ancestor with Get3d (Fig. S1).

To investigate the conservation of Get3d in photosynthetic bacteria specifically, we quantified the number of sequenced genomes that contain Get3 proteins with the characteristic αCD in various phyla (Fig. S2*D*). Get3d is completely conserved in cyanobacteria and green sulfur and nonsulfur bacteria, which do not contain another Get3-like homolog. In purple bacteria, many families contain species that encode a cytoplasmic Get3 homolog, whereas only a single purple nonsulfur bacterium encodes a Get3d homolog. Furthermore, the number of Get3d homologs encoded by representative species from these phyla was determined (Fig. S2*E*). While some species contain no Get3d proteins, others contain multiple copies of Get3d in their genome, such as *Chlorobium chlorochromatii*, which contains five copies of Get3d. This could be due to whole genome duplication events (34) and horizontal gene transfers (35, 36), which are integral to the evolution of protein families and homologs (37). As the availability of sequenced genomes from photosynthetic bacteria increases, a broader depth of information about the evolution and conservation of Get3d in photosynthetic bacteria can be learned.

We next consider the distribution of the Get3 homologs across plants. Using our phylogenetic information, we assigned each Get3 to either the a, b/c, or d group, we can correlate the taxonomic distribution of each group across Viridiplantae (Figs. 1*C* and S3). The tree is collapsed at the level of taxonomic genus to minimize errors resulting from uneven genome annotation; however, in some cases, poor annotation within a genus may preclude the ability to identify a given homolog. The results suggest that at least one protein from each of the Get3a and Get3b/c groups is present across all plant genomes. Get3d proteins are found across land plants (*e.g.*, mosses, grasses, and eudicots) yet are completely missing in single-celled plants, the blue branch in Figures 1*C* and S3. Given that the nearest relative of Get3d is from a cyanobacteria and plants are derived from an endosymbiotic event that led to a chloroplast (10, 38, 39), the absence of Get3d in single-celled plants suggests gene loss in the corresponding taxa.

### Subcellular localization of Get3d

The cellular localization of *At*Get3d has not been experimentally determined. Like many essential genes from the ancestral cyanobacteria, Get3d acquired a chloroplast targeting signal during the endosymbiotic transfer of genes into the plant genome (10, 38, 39). Computational methods support this predicting that *At*Get3d is localized to the chloroplast stroma (∼91% likelihood) with a small probability (∼5%) of it being localized to the thylakoid space (40).

**Figure 2:**
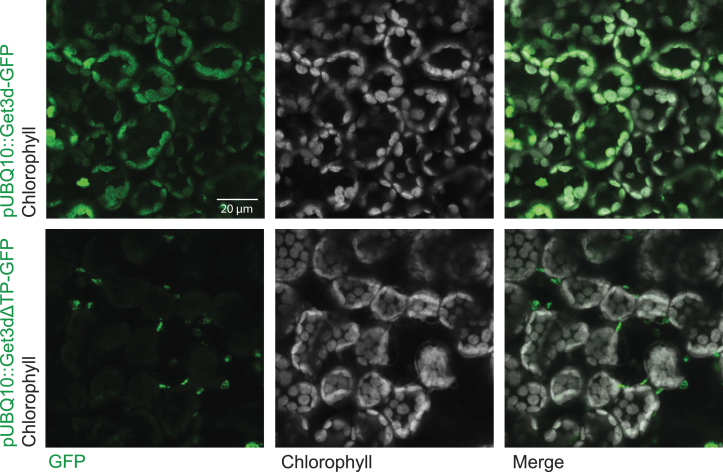
**Cellular localization of *At*Get3d.** Confocal microscopy images of *Nicotiana benthamiana* leaf mesophyll infiltrated with *Agrobacterium tumefaciens* harboring T-DNA plasmids containing *At*Get3d-GFP with (*top*) and without (*bottom*) the chloroplast transit peptide (TP). Get3d is expressed under the control of the pUBQ10 promoter. Scale is shown. *At*Get3d, *Arabidopsis thaliana* Get3d.

To experimentally confirm the localization of *At*Get3d, we employed *Agrobacterium*-mediated expression of *At*Get3d in *Nicotiana benthamiana* leaves (41). Constructs were generated with GFP appended to the Get3d gene, with or without the predicted chloroplast transit peptide, as a C-terminal fusion. This allowed us to monitor the localization of expressed Get3d after *Agrobacterium*-mediated insertion into the *Nicotiana* genome. Upon infiltration, the tobacco leaves were monitored by fluorescence microscopy. For the full-length Get3d gene that contained the transit peptide, the fluorescence colocalized with the intrinsic chlorophyll autofluorescence, indicating that *At*Get3d localizes to the chloroplast (Fig. 2). Localization to the stroma *versus* thylakoid lumen could not be distinguished here. For Get3d lacking the transit peptide, the GFP signal gave a pattern of distinct puncta not associated with a clear subcellular structure in the mesophyll cells. The puncta are not consistent with the typical localization of GFP, the cytosol and nucleus, in these cells (42). These results confirm the predictions of a functioning chloroplast targeting signal.

### Crystal structures of photosynthesis-associated Get3d

**Figure 3:**
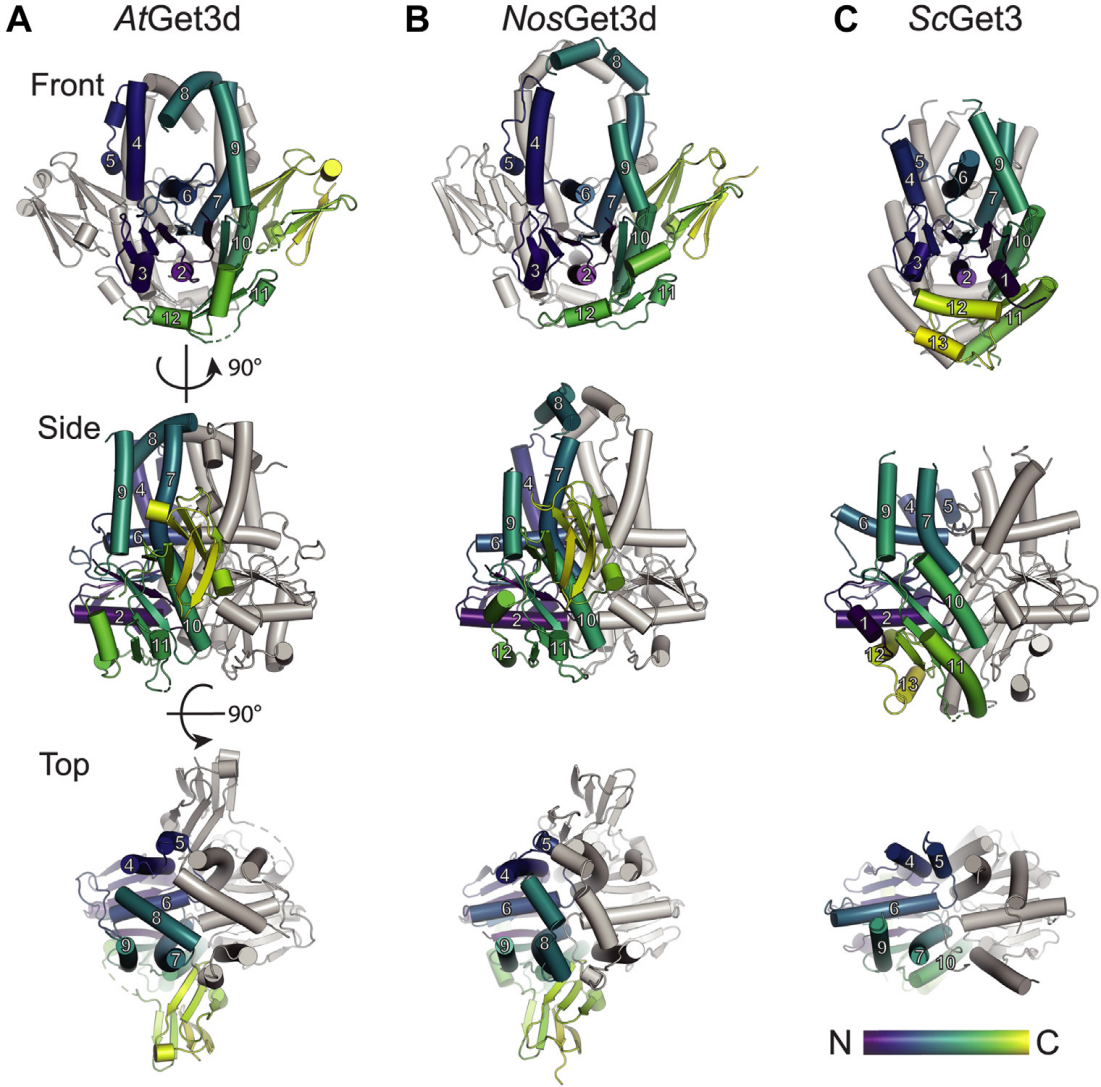
**Structures of *At*Get3d and *Nos*Get3d.***Front*, *side*, and *top* views of the structure of (*A*) *At*Get3d (Protein Data Bank [PDB] ID: 8ELF), (*B*) *Nos*Get3d (PDB ID: 8EGK), and (*C*) the closed conformation of yeast Get3 (*Sc*Get3, PDB ID: 2WOJ). For each, one monomer is shown in *Viridis*, and the other is shown in *gray*. Transmembrane domains (TMDs) are numbered from N to C terminus for reference based on the fungal Get3 structure. *At*Get3d, *Arabidopsis thaliana* Get3d; *Nos*Get3d, *Nostoc* sp. Get3d; *Sc*Get3, *Saccharomyces cerevisiae* Get3.

The significant differences in Get3 sequences hint at unique structural features of the photosynthetic homologs, motivating us to solve the crystal structure of *At*Get3d (Figs. 3*A* and S4*A*). We generated a construct without the chloroplast transit peptide (Δ1–57) that purified as a single peak *via* size-exclusion chromatography, consistent with a homodimer. While the sequence suggested missing residues for ATP binding, only crystals grown in the presence of ADP resulted in diffraction. Final crystals, grown by sitting drop in 50 mM sodium cacodylate (pH 5.47), 50 mM lithium sulfate, and 30% PEG-4000, were frozen with the addition of 30% glycerol as a cryoprotectant, and a complete native dataset was collected to 2.0 Å resolution in the space group P 1 2_1_ 1. The closest homolog, with 31% sequence identity, in the Protein Data Bank (PDB) is the cyanobacterial *Nos*Get3d (PDB ID: 3IGF) (43, 44) from *Nostoc* sp. PCC 7120 (also referred to as *Anabaena* sp. PCC 7120). The *Nos*Get3d homodimer was used as a search model to obtain phases by molecular replacement. As anticipated, the structure contained a homodimer in the asymmetric unit. The structure was refined to an *R*-factor of 0.22 and free-*R*-factor of 0.26 (crystallographic statistics in Table S1). No density for nucleotide was visible in the putative active site; however, there was clear density for an inorganic phosphate and an Mg^2+^ ion (Fig. S4*B*). Residues 250 to 260, 330 to 331, and 378 to 382 in monomer A and residues 252 to 261 and 380 to 384 in monomer B could not be resolved in the density.

Like structures of Get3 homologs, *At*Get3d is a homodimer with a core nucleotide-binding domain and an α-helical client-binding domain (CBD) (Figs. 3*A* and S4*A*) (32, 45). A structural alignment suggests that, after *Nos*Get3d (Fig. 3*B*), *At*Get3d is most similar to the closed conformation of yeast Get3 (45) (Fig. 3*C*). Consistent with our bioinformatic analysis, *At*Get3d has an αCD at its C terminus, which is unique to the Get3d clade (Fig. S4*A*). The P-loop, switch I, and switch II that define the nucleotide-binding domain are conserved in this subgroup, whereas the A-loop, which recognizes the adenosine of the substrate ATP, is missing, as predicted from the sequence (Figs. 1*B* and S5, *A*–*C*). *At*Get3d also lacks the helix that would contain the CXXC motif and, expectedly, no bound Zn^2+^ is observed (Figs. 1*B* and S5*D*). As opposed to the groove seen in fungal Get3 structures, the CBD of *At*Get3d is a chamber (Fig. S4*A*) (32, 45).

Upon inspection, additional density was visualized in the chamber (Fig. S6*A*) that could not be explained by protein or solvent. Native mass spectrometry was performed, and analysis confirmed that *At*Get3d is a dimer and that it copurifies with small molecules (∼750 ± 140 Da) (Fig. S6, *B* and *C*). Although the mass of these adducts is consistent with phospholipids (46), it is not possible to assign the exact lipid given the mass and associated error. Considering these results, the density, and the properties of the binding site, a phosphatidic acid was built into this density (Fig. S6, *A* and *D*). The aliphatic chains fit the two tubes of density, whereas the phosphate head group forms a salt bridge with R176, R182, and a possible H-bond with Q136 (Fig. S6*A*). The general features are found in the *Nos*Get3d structure, yet it is unlikely that this is primarily a lipid-binding site as the charged residues are not conserved (Fig. S6*E*).

### A refined structure of *Nostoc* sp. Get3d

In *At*Get3d, the α-helical CBD is enclosed by additional helices not seen in the deposited Get3d structure from *Nostoc* sp. (Figs. 3*A* and S7*A*). We viewed the electron density for the *Nostoc* sp. homolog using the deposited structure factors for PDB ID: 3IGF (43). With this map, we could clearly identify additional density consistent with the helices of *At*Get3d that enclosed the CBD. We built into this density adding 41 residues total and further refined the *Nos*Get3d structure (Fig. S7, *B*–*D*) with refinement statistics in Table S1.

Overall, *Nos*Get3d is structurally very similar to the *At*Get3d structure (backbone RMSD = 2.33 Å). As with *At*Get3d, *Nos*Get3d has some unidentified densities in the hydrophobic chamber (Fig. S6*F*). One prominent difference is that the helices enclosing the CBD in *Nos*Get3d are further from the bottom of the chamber than in *At*Get3d, resulting in a larger hydrophobic chamber (Fig. 3, *A* and *B*).

### The αCD

**Figure 4:**
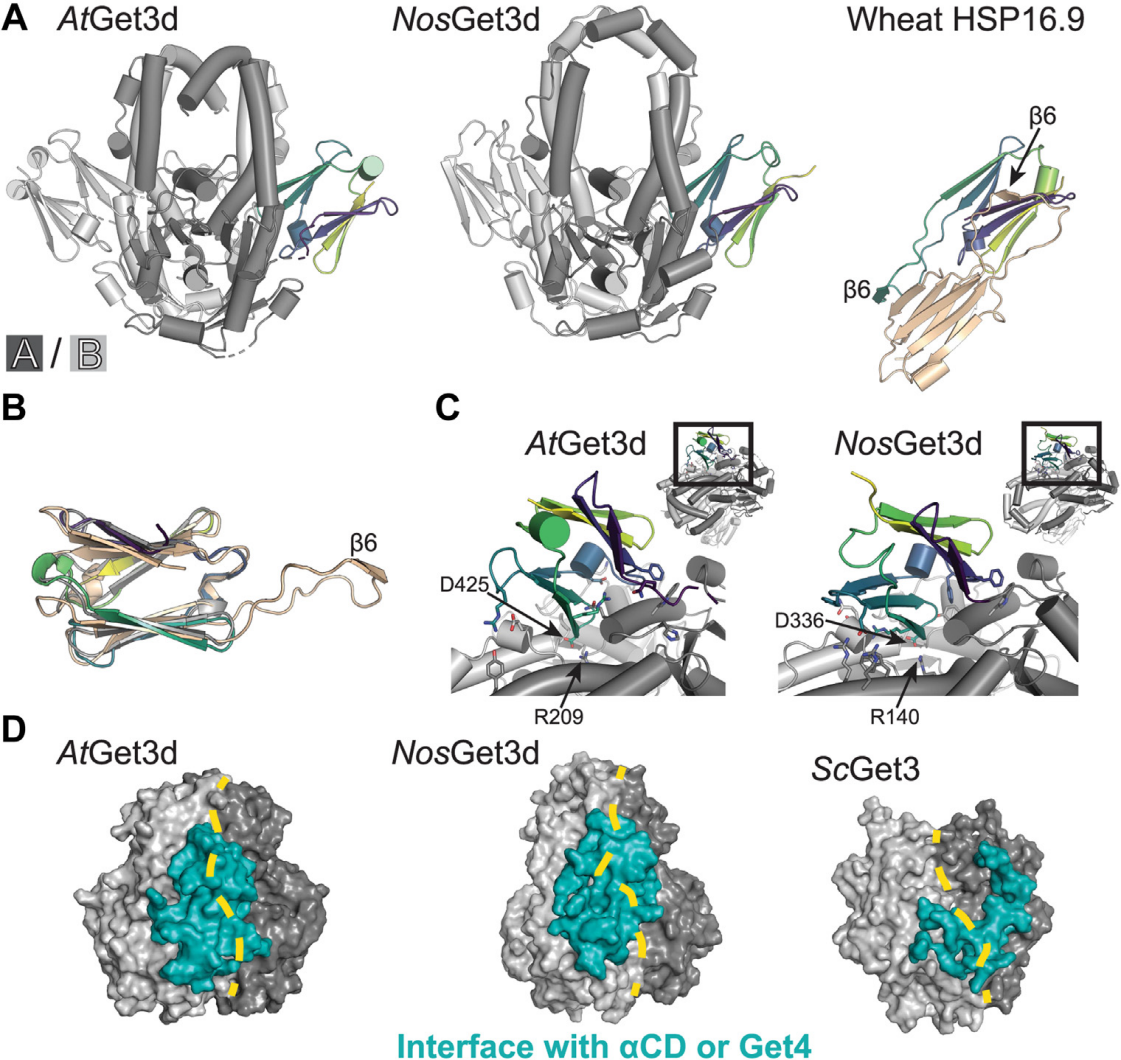
**Structural analysis of the α-crystallin domain (αCD) of Get3d.***A*, front view of the structure of *At*Get3d and *Nos*Get3d and the αCD of wheat HSP16.9 (Protein Data Bank [PDB] ID: 1GME) (from *left* to *right*). Get3d colored with monomer A αCD (*Viridis*), monomer A Get3 domain (*dark gray*), and monomer B (*light gray*). HSP16.9 colored monomer A (*Viridis*) and monomer B (*wheat*). *B*, alignment of the αCD of *At*Get3d (*Viridis*), *Nos*Get3d (*gray*), and wheat HSP16.9 (*wheat*). *C*, residues involved in electrostatic and hydrophobic interactions at the interface of the αCD of *At*Get3d (*left*) and *Nos*Get3d (*right*) shown as *sticks* with discussed residues labeled. For each, the region highlighted is shown in the full structure above. Colored as in (*A*). *D*, the interaction surface of the αCD of *At*Get3d and *Nos*Get3d with the Get3 domain and yeast Get3 (*Sc*Get3) with Get4/5 (PDB ID: 4PWX) (from *left* to *right*) showing the Get3 domains (*dark and light gray*), interaction surface (*dark teal*), and interface between the two Get3 domains (*dotted yellow line*). *At*Get3d, *Arabidopsis thaliana* Get3d; *Nos*Get3d, *Nostoc* sp. Get3d; *Sc*Get3, *Saccharomyces cerevisiae* Get3.

A distinctive feature of Get3d is the αCD at its C terminus (Fig. 4*A*). This domain has the hallmark α-crystallin fold, a compact β-sandwich composed of seven antiparallel β-strands (Fig. 4, *A* and *B*) (47, 48). While the fold is conserved, this domain has low sequence similarity to other αCDs and the related small heat shock proteins (sHSPs) and is missing typical features important to αCD dimerization and oligomerization, such as the loop containing β6 found in most plant, yeast, and bacterial αCD/sHSPs (Fig. S8). We have found no evidence of either the αCD dimers or higher order oligomers that are found for most αCDs.

In both structures, the αCD sits at the interface of the two Get3 monomers with electrostatic and hydrophobic interactions to both monomers (Figs. 4*C* and S9*A*). The interface buries ∼1270 Å^2^ in *At*Get3d and ∼1280 Å^2^ in *Nos*Get3d. The αCD occupies the same binding site as Get4 on cytoplasmic Get3 with a comparable buried surface (∼1320 Å^2^, PDB ID: 4PWX) (Figs. 4*D* and S9*A*) (49). Overall, the two Get3d interfaces have similar properties, yet the specific electrostatic and hydrophobic interactions are different. Some residues are conserved, such as *At*Get3d R209 and D425, which form a salt bridge across the interface (Fig. 4*C*). In *At*Get3d, the loop connecting the N terminus of the αCD to the rest of the protein is partially disordered (Fig. 4*A*). Disruption of the αCD interface would likely result the αCD being loosely associated to the rest of the protein. It is interesting to speculate that under some conditions, this domain could be exposed.

There are a few additional features of interest related to the αCD interface with the rest of Get3d. First, in *At*Get3d, a salt bridge formed between K107 in the Get3 domain to E404 in the αCD of the opposite monomer is reminiscent of a salt bridge between the homologous yeast Get3 K69 to D74 of Get4 (Fig. S9*B*). In the cytoplasm, this interaction regulates Get3 ATPase activity (49). Here, this interaction could be important for regulating communication between the αCD and the active site, although it is not conserved in *Nos*Get3d. A second surprising feature is that both Get3d homologs contain a conserved cis-proline in the loop before helix *α*11 (340 in *At*Get3d and 265 in *Nos*Get3d), which is not present in cytoplasmic Get3 proteins (Fig. 3).

### Get3d as an ATPase

**Figure 5:**
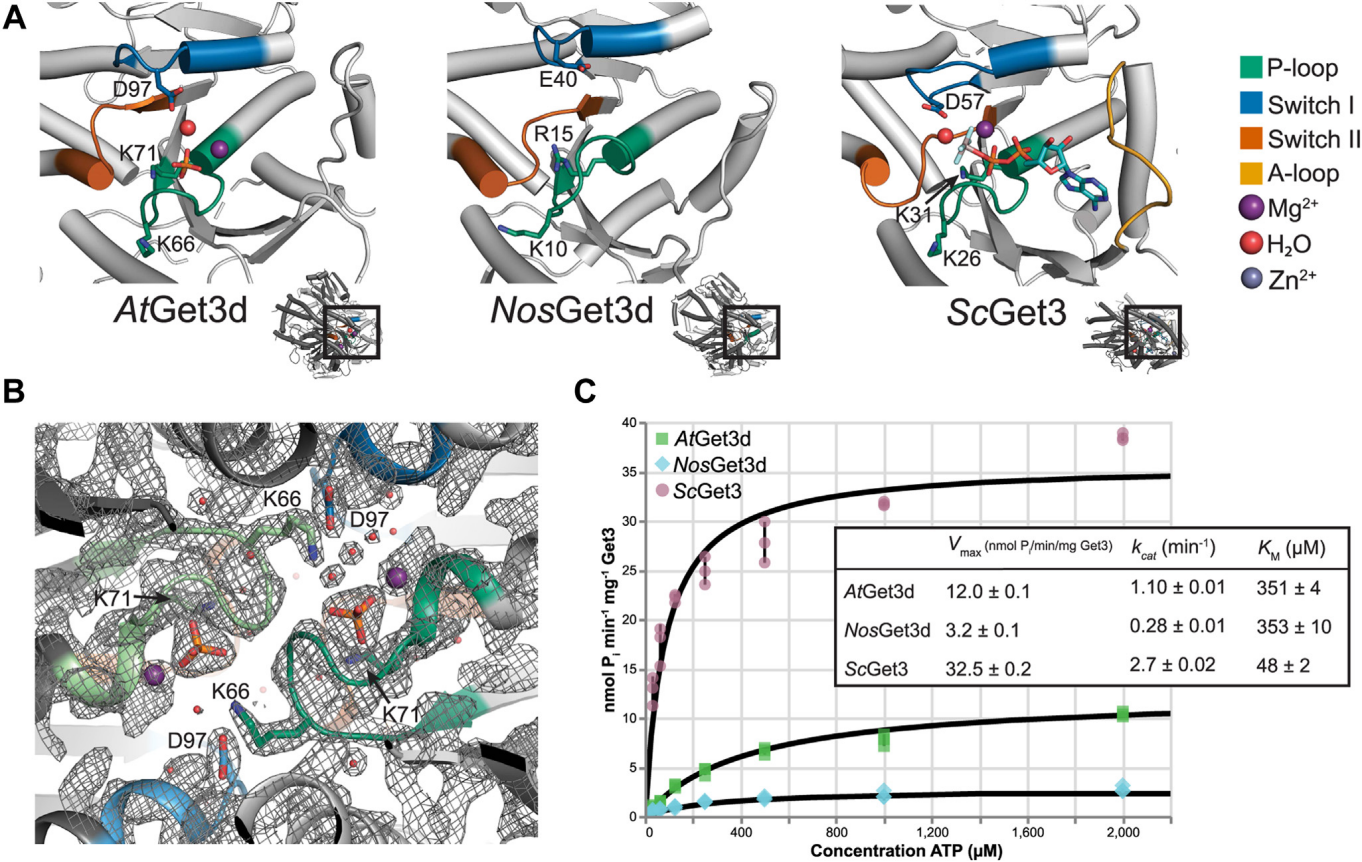
**ATPase activity of Get3d.***A*, the active site and signature Get3 features of a monomer of *At*Get3d, *Nos*Get3d, and the closed conformation of yeast Get3 (*Sc*Get3, Protein Data Bank [PDB] ID: 2WOJ) (from *left* to *right*). Signature Get3 features are colored P-loop (*green*), switch I (*blue*), switch II (*orange*), A-loop (*yellow*), Mg^2+^ (*purple sphere*), Zn^2+^ (*gray sphere*), and H_2_O (*red sphere*). ATP and P_i_ are shown as *sticks*. For each, the region highlighted is shown on the *right* in the full structure. *B*, 2*F*_O_–*F*_C_ electron density (*light gray mesh*) in the active site of *At*Get3d contoured at 1.5*σ*. Discussed residues are shown as *sticks*. Colored as in (*A*) showing both P-loop (*dark and light green*) and switch I motifs (*dark and light blue*). *C*, ATPase activity of *At*Get3d, *Nos*Get3d, and yeast Get3 (*Sc*Get3) in nmol P_i_ min^−1^ mg^−1^ Get3 *versus* concentration ATP (micromolar) determined with the EnzCheck Phosphate Assay. Analyzed using ICEKAT (99) with kinetic constants reported. Standard deviation is shown as error bars. For *Sc*Get3, previously reported *k*_cat_ is 1.3 ± 0.4 min^−1^ and *K*_*M*_ is 37 ± 6.7 μM (6). *At*Get3d, *Arabidopsis thaliana* Get3d; *Nos*Get3d, *Nostoc* sp. Get3d; *Sc*Get3, *Saccharomyces cerevisiae* Get3.

Both *At*Get3d and *Nos*Get3d retain components required for ATPase activity including the P-loop (Walker A motif) that recognizes the α- and β-phosphates of the substrate ATP, as found in fungal Get3 (Fig. 5, *A* and *B*) (32, 45). Get3 belongs to the Mrp/MinD subfamily of the SIMIBI class of NTPases, which are characterized by having an intradimeric (or “deviant”) Walker A motif (28, 31, 33, 50). The canonical Walker A motif contains a conserved lysine (GxxGxG**K**[ST]) that mediates phosphate binding. The intradimeric Walker A motif contains a second conserved lysine (G**K**GGhGK[ST]) that reaches across the dimer interface when ATP is bound to facilitate catalysis. In cytoplasmic Get3 proteins, the intradimeric Walker A lysine stabilizes the accumulation of negative charge that builds up in the active site during the water-mediated nucleophilic attack on the γ-phosphate (32, 45). This lysine, and presumably its catalytic role, is conserved in Get3d (Fig. 5, *A* and *B*). The canonical Walker A lysine, which is broadly conserved in P-loop NTPases (28) including Get3 (32, 45), points toward the β-phosphate within the same monomer. In the *At*Get3d crystal structure, the bound inorganic phosphate correlates to the β-phosphate in the ATP-bound Get3 structures and is partially coordinated by this lysine (Fig. 5, *A* and *B*) (5, 45). While the lysine is conserved in *At*Get3d, it is unexpectedly an arginine in *Nos*Get3d (Figs. 1*B* and 5, *A* and *B*), which is distinct from most cyanobacterial Get3d homologs and would suggest that in this organism there has been significant evolutionary drift.

For the catalytic switch loops, switch I is structurally conserved, whereas switch II is conserved at both the sequence and structural levels (Figs. 1*B* and 5, *A* and *B*). In P-loop NTPases, switch I and switch II couple structural rearrangements to the presence of the ATP γ-phosphate (28). In cytoplasmic Get3 proteins, it has been shown that the highly conserved aspartate in switch I coordinates a water, helping to align the water for nucleophilic attack on the γ-phosphate (Fig. 5*A*) (45). This aspartate is conserved in the Get3d family, with *Nos*Get3d uniquely having a glutamate at this position, further supporting its evolutionary drift (Figs. 1*B* and 5*A*). Notably, in *At*Get3d, the water is in a slightly different position likely because of the slightly shorter switch I and the presence of an inorganic phosphate instead of a nucleotide (Fig. 5, *A* and *B*). The sequence and structural conservation of these features suggests that some nucleotide-dependent structural rearrangements could occur in Get3d.

Both *At*Get3d and *Nos*Get3d are missing features that select for the adenosine nucleoside. Rather than an asparagine in strand β7, which specifically selects for the adenine base (32, 45), both *At*Get3d and *Nos*Get3d have an isoleucine (Fig. S5, *A*–*C*). Importantly, the A-loop present in cytoplasmic Get3 proteins, which interacts with both the adenine and the ribose, is completely missing in the Get3d structures described here (Figs. 1*B*, 5, *A* and *B*, and S5, *A*–*C*). This suggests that Get3d may not be specific for ATP.

In addition, both *At*Get3d and *Nos*Get3d are missing the Zn^2+^-coordinating CXXC helix (Figs. 1*B* and S5*D*). The Zn^2+^ acts as a pivot point for the conformational changes coordinated with the ATPase cycle. While there are some examples of Get3 proteins missing the CXXC motif (Fig. 1*B*) (20, 23, 51, 52), it is not known in these cases how conformational changes are coupled to ATP hydrolysis, which is critical in the targeting cycle (5). As Get3d adopts a closed conformation in the absence of nucleotide, there are likely unique conformational changes associated with these proteins.

As Get3d retains most of the components required for nucleotide hydrolysis, it is important to establish that Get3d is an NTPase. To investigate this, the ATPase activities of *At*Get3d and *Nos*Get3d were determined by monitoring the phosphate produced by Get3d in a spectrophotometric assay (Fig. 5*C*). Both *At*Get3d and *Nos*Get3d were found to have ATPase activity, with a *V*_max_ of 12.0 ± 0.1 and 3.2 ± 0.1 nmol P_i_/min/mg Get3 and *k*_cat_ of 1.10 ± 0.01 and 0.28 ± 0.01 min^−1^, respectively (Fig. 5*C*). Notably, *At*Get3d and *Nos*Get3d have similar affinities for ATP with a *K*_*M*_ of 351 ± 4 and 353 ± 10 μM, respectively (Fig. 5*C*). To compare this, the ATPase activity of yeast Get3 was determined, a *V*_max_ of 32.5 ± 0.2 nmol P_i_/min/mg Get3, *k*_cat_ of 2.7 ± 0.02 min^−1^, and a *K*_*M*_ of 48 ± 2 μM (Fig. 5*C*), consistent with values previously observed (6, 45). Both *At*Get3d and *Nos*Get3d have a lower affinity for ATP and a slower maximum velocity compared with yeast Get3, likely because of the structural differences in the active site and the lack of the A-loop, which facilitates nucleotide binding.

### The hydrophobic chamber and binding to a TA protein client

**Figure 6:**
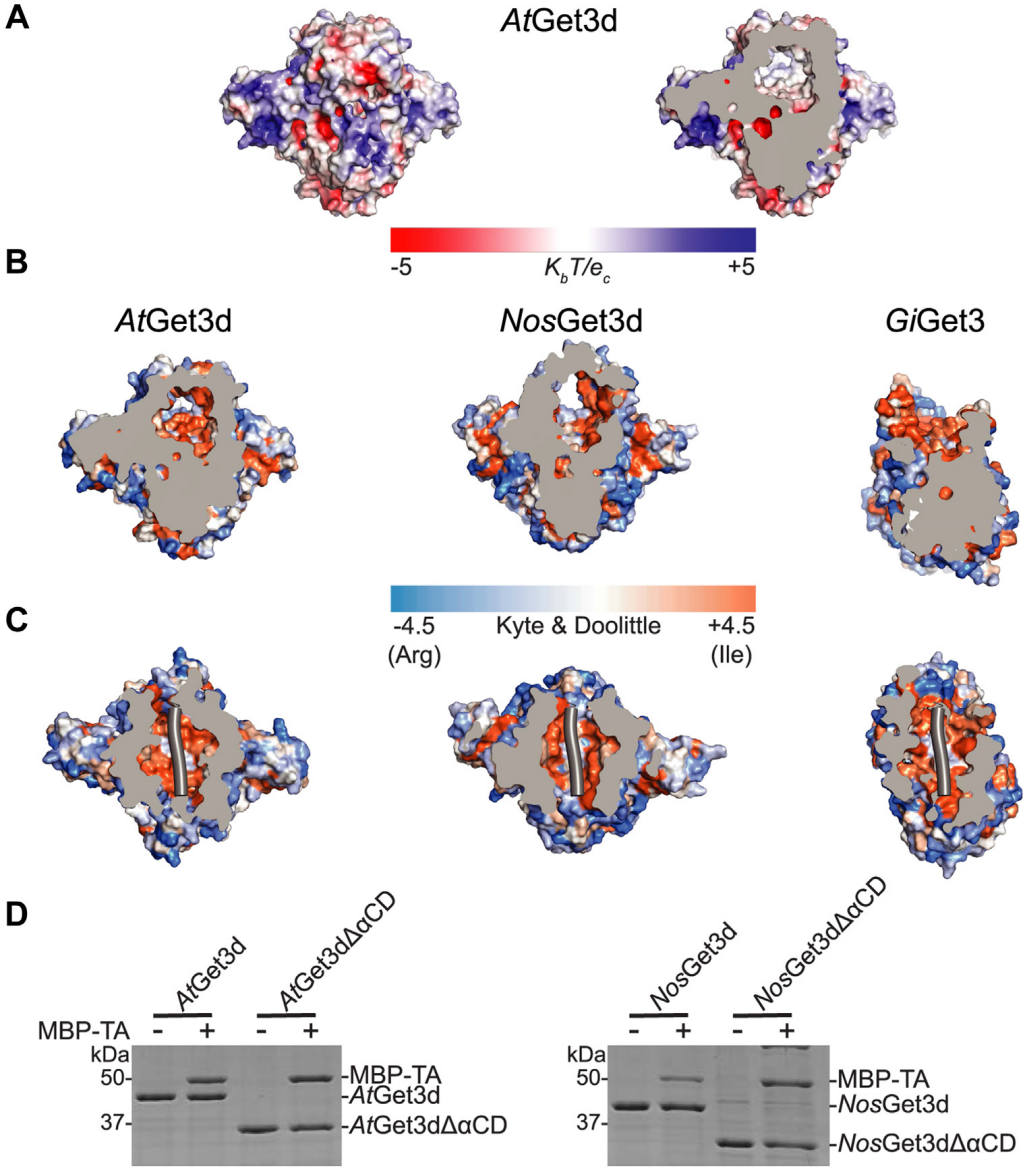
**The hydrophobic chamber of Get3d.***A*, full (*left*) and slice view (*right*) surface electrostatic potential of *At*Get3d as for Fig. S10*B*. *B*, slabbed view of accessible surface of *At*Get3d, *Nos*Get3d, and *Gi*Get3 (Protein Data Bank [PDB] ID: 7SQ0) (from *left* to *right*) colored by hydrophobicity using the Kyte and Doolittle scale. *C*, top–down slice view of the surface hydrophobicity of *At*Get3d, *Nos*Get3d, and *Gi*Get3 in complex with the transmembrane domain (TMD) of the yeast TA protein Bos1 (PDB ID: 7SQ0) (from *left* to *right*). After aligning the three structures, Bos1 is overlaid on the *At*Get3d and *Nos*Get3d structures. Scale as in (*B*). *D*, *in vitro* TA protein capture assays in which His-tagged *At*Get3d and *Nos*Get3d with and without the α-crystallin domain (αCD) are expressed in the absence or the presence of a maltose-binging protein (MBP)–tagged yeast TA protein Sbh1. After purification by nickel affinity chromatography, the eluate is analyzed by Coomassie-stained SDS-PAGE. *At*Get3d, *Arabidopsis thaliana* Get3d; *Gi*Get3, *Giardia intestinalis* Get3; *Nos*Get3d, *Nostoc* sp. Get3d; TA, tail-anchored.

The CBD of *At*Get3d is comprised of 10 amphipathic helices (Figs. 3*A* and S4*A*), which form a hydrophobic chamber (Fig. 6, *A* and *B*). Two crossing helices (α6 using the same numbering convention described previously (45)) form the bottom of the chamber, the sides are formed by four helices (α4, α5, α7, and α9), and the chamber is enclosed on the top by two additional helices (α8) (Fig. 3*A*). The inside of the chamber is characterized by hydrophobic and uncharged amino acids, which would be expected for a binding site of a TMD (Fig. 6, *A* and *B*). There is sufficient volume in the chamber to accommodate a TMD of ∼22 amino acids. To visualize this, we aligned Get3d to the structure of *Giardia intestinalis* Get3 (*Gi*Get3) in complex with the yeast TA protein Bos1 (PDB ID: 7SQ0) (5). Here, the TMD of Bos1 fits easily into the chamber (Fig. 6*C*). The chamber of *Nos*Get3d is similar, albeit slightly larger (Figs. 6, *B* and *C* and S10*A*). The chamber is a unique feature of Get3d, in stark contrast to the hydrophobic groove seen in closed fungal Get3 structures (Figs. 3*C* and S10, *B* and *C*) and demonstrates a mechanism for closing reminiscent of the *Giardia* Get3–TA complex (5, 45, 53). As we know that this chamber can accommodate a TA protein, it will be interesting to see how this domain rearranges.

To determine the TA protein binding capability of *At*Get3d and *Nos*Get3d, His_6_-tagged Get3d constructs were expressed alone or coexpressed with a maltose-binding protein (MBP)–tagged yeast TA protein, Sbh1. Capture assays were performed by passing the lysate over immobilized metal affinity chromatography followed by elution with imidazole. Stable complexes were observed by Coomassie-stained SDS-PAGE (Fig. 6*D*). Both constructs were able to capture the TA protein, confirmed by Western blot (Fig. S10*D*). The specificity of the Get3d and TA interaction was confirmed by repeating the procedure with MBP-TA alone, and as expected, no binding of MBP-TA was observed (Fig. S10*E*). The ability of Get3d to form a complex with a TA protein suggests that Get3d may be involved in TA protein targeting, or, at a minimum, bind TA proteins to protect them from the aqueous environment as a general chaperone. As αCDs have been shown to bind to unfolded proteins (47, 48, 54), we wanted to see if the αCD was required for TA protein complex formation. Capture assays were performed in a similar manner for both constructs with the αCD removed. These were also able to capture the TA protein (Figs. 6*D* and S10*D*), revealing that the αCD is not required for TA protein binding.

## Discussion

In eukaryotes, the efficient and precise insertion of membrane proteins is an imperative step for their accurate function in various organelles (1, 55, 56). Errors in targeting may lead to mislocalization of these proteins, which can result in unfavorable cellular effects. Recent work explores the GET pathway in plants, with all components of the GET pathway excluding Get5 and Sgt2 having been identified (18, 19, 20, 23, 57). However, a striking difference in plants compared with other eukaryotes is the presence of multiple paralogs of Get3 (8, 17, 19). In *A. thaliana*, four paralogs of Get3 exist, termed Get3a–d. This study is the first to characterize Get3d, a distinct homolog that is conserved across a few billion years of the evolution of photosynthesis from bacteria to plants.

Overall, some Get3d proteins are more similar in sequence to the cytoplasmic Get3 proteins, whereas others are more diverged (Fig. 1*B*) suggesting distinct evolutionary paths. For example, Get3d proteins from representative green sulfur and green nonsulfur bacteria, along with *Synechocystis* sp. Sll0086, contain a highly conserved switch I and A-loop, whereas *At*Get3d, *Nos*Get3d, and *Synechocystis* sp. Slr1794 do not. It will be interesting to investigate the differences between the two *Synechocystis* sp. Get3d proteins as one is more similar to *At*Get3d and *Nos*Get3d than its paralog, which has features closer to cytoplasmic Get3 proteins implying distinct roles.

Catalytic residues show some interesting variability in Get3d. The canonical Walker A lysine, which coordinates the nucleotide β-phosphates, is broadly conserved in P-loop NTPases (28). For Get3d, this lysine is conserved throughout angiosperms, such as *A. thaliana*. Green sulfur (*Chlorobiaceae*) and green nonsulfur (*Chloroflexi*) bacteria also have a lysine at this position (Fig. 1*B*). Certain cyanobacterial Get3d proteins have replaced this with an arginine, which is unique to Get3d proteins that appear early in the cyanobacterial lineage, including simple filamentous cyanobacteria, such as some *Pseudanabaena* species, *Leptolyngbya*, *Halomicronema*, and several clades of synechococcalean cyanobacteria (38, 58). Based on this evidence, the Walker A lysine may have mutated early in the cyanobacterial lineage, whereas green sulfur and nonsulfur bacteria and angiosperms retained the lysine. The catalytic aspartate in Get3, D57 in yeast, coordinates water and primes it for nucleophilic attack of the γ-phosphate of ATP (32, 45). This residue is highly conserved in Get3d proteins; however, it is a glutamate in *Nos*Get3d, which may contribute to its lower *V*_max_ (Figs. 1*B* and 5, *A* and *C*).

When examining the structure of Get3d in detail, it is unclear if it should be able to hydrolyze ATP. The variations in Get3d around the active site, such as the slightly shorter loop in switch I and the absence of the A-loop, may preclude Get3d from hydrolyzing ATP and producing the conformational changes that are coupled to TA protein targeting in fungal Get3 (32, 45). Our data reveal that both *At*Get3d and *Nos*Get3d are active ATPases (Fig. 5*C*). The decreased K_*M*_ of Get3d for ATP when compared with yeast Get3 is likely due to the absence of the A-loop (Figs. 5*A* and S5, *A*–*C*).

Notably, the ATPase cycle and conformational changes of Get3, which regulate protein targeting, are inextricably linked (4, 5, 6). As Get3d can hydrolyze ATP (Fig. 5*C*), Get3d likely adopts additional conformations relative to the structures presented here. The structures here are most similar to the closed conformation of Get3, yet they do not have either ATP or an ATP analog bound. The closed conformation of Get3 depends on a bound ATP or ATP analog (*e.g.*, ADP–aluminum fluoride closed yeast dimer [PDB ID: 2WOJ] (45)); therefore, the missing nucleotide in the Get3d structures is surprising. For the *At*Get3d structure, the requirement for ADP in the crystallization condition suggests that the phosphate bound in the active site may support a conformational change and could act in a regulatory manner similar to that of ATP binding and hydrolysis. Studies have shown that the concentration of inorganic phosphate in the chloroplast stroma changes drastically with changes in light conditions across the day/night cycle (59), and it is conceivable that this fluctuation may regulate Get3d in some manner.

An interesting feature of Get3d is the presence of a hydrophobic chamber in the closed state instead of a client-binding groove as seen in closed fungal Get3 structures, suggesting this may be the default state for Get3d (Figs. 6, *A*–*C* and S10, *A*–*C*). Density in the hydrophobic chamber of *At*Get3d consistent with a phospholipid (Fig. S6, *A* and *D*) is unsurprising as the stable hydrophobic chamber may nonspecifically carry the lipid through purification, although it is possible that Get3d may have a role in lipid binding *in vivo*. Phospholipids are major components of the cytoplasmic membrane in both cyanobacteria and *Escherichia coli* (60). The head group of the modeled phosphatidic acid forms specific interactions (Fig. S6*A*); however, only the positive charge of the *At*Get3d R182 is conserved in Get3d proteins overall (Fig. S6*E*). The acyl chain lining the bottom of the hydrophobic chamber is more ordered than the other, which extends into the chamber, likely because of strong interactions with the hydrophobic residues of the groove (Fig. S6, *A* and *D*). Further studies will be necessary to determine if there is a physiological role for lipid binding.

The most parsimonious model is that Get3d plays a role in TA protein targeting similar to the cytoplasmic Get3 proteins (6, 17, 45). In our TA protein capture assays, both *At*Get3d and *Nos*Get3d were able to form a stable complex with a TMD (Figs. 6*D* and S10*D*), consistent with the structure. Further studies will be necessary to demonstrate a direct role in TA protein targeting. If Get3d does participate in TA protein targeting, it would necessitate new partners as no other GET pathway components have been identified in the chloroplast (18, 20, 23, 57). These Get3d partners would likely be conserved in photosynthetic bacteria as well.

The presence of an αCD appended to the C terminus is a unique feature of Get3d proteins (Fig. 4*A*). Many α-crystallins/sHSPs act as ATP-independent chaperones by binding to unfolded proteins to protect cells from damage because of protein aggregation (54). Because the αCD of Get3d is not required for TA protein binding (Figs. 6*D* and S10*D*), the role of the αCD is unclear. The presence of the αCD may suggest that *At*Get3d acts as a general chaperone in a manner similar to α-crystallins/sHSPs. While the αCD of Get3d maintains the overall fold of α-crystallins/sHSPs, it lacks the features characteristic of oligomerization. Thus, if the αCD of Get3d does oligomerize, it would require novel architectures. As the interface between the Get3 and αCD of Get3d is similar to that of yeast Get3 and Get4 (Fig. 4*D*), it is possible that the αCD of Get3d stabilizes the closed conformation and/or acts in a regulatory manner similarly to how yeast Get4 regulates ATP hydrolysis by Get3 (6, 49). Another possible function could be that the αCD binds and stabilizes the N-terminal soluble domains of specific TA protein clients; however, not all proteins with αCDs act as chaperones, thus care must be taken when classifying new αCD-containing proteins (47, 48, 61). Further investigation is needed to shed light on the significance of αCD of Get3d.

The absence of the CXXC motif and its coordinated Zn^2+^ ion in Get3d is an important distinction (Figs. 1*B* and S5*D*). While conserved in most Get3 proteins, its presence is not necessary for Get3 activity in all cases (20, 23, 51). Of note, *At*Get3a, the cytoplasmic Get3 that targets TA proteins to the ER in *A. thaliana*, also lacks the CXXC motif (Fig. 1*B*) (20, 23). In addition to a role in dimerization, the CXXC motif has also been implicated as modulating a secondary function of Get3 as a general chaperone regulated by oxidation (62). Get3d may bypass this requirement to act as a general chaperone.

Evidence supports that *A. thaliana* has two Get3 paralogs localized to the chloroplast: Get3b and Get3d (14, 20). While *At*Get3d appeared early in the evolution of photosynthesis, *At*Get3b is first found with the appearance of chloroplasts, suggesting it was a newly acquired role in plants. A possible role is that *At*Get3b and *At*Get3d are both involved in TA protein targeting with different substrate specificities or different destination membranes (thylakoid *versus* inner envelope membrane). This is an exciting hypothesis, as *At*Get3b was shown to interact with the thylakoid membrane protein *At*SECE1 but not the inner envelope membrane *At*SECE2 (14). While previous work has shown that *At*Get3b localizes to the chloroplast stroma specifically (14), we were unable to distinguish between the stroma and thylakoid lumen here. Thus, the possibility that *At*Get3b functions in the stroma and *At*Get3d functions in the thylakoid lumen cannot be ruled out. *At*Get3b and *At*Get3d may also act in different tissues, in different plastid types (*e.g.*, chloroplast, leucoplast, and chromoplast) (63), or during different stages of development. As they are both conserved across plants, it will be necessary to determine the roles of *At*Get3b and *At*Get3d.

If Get3d plays a role in TA protein targeting in chloroplasts, possible clients include several essential chloroplast-encoded proteins such as multiple photosystem I and II reaction center components and several cytochrome *b*_*6*_*f* proteins (64). The pool of substrates may also include nuclear-encoded TA proteins such as SECE1 and SECE2. Many of these proteins are conserved across all photosynthesis and represent an interesting pool of possible Get3d substrates.

The deep evolutionary connection between the Get3d fold and photosynthesis, while correlative, does not address the function of Get3d. Clearly, the preservation of its presumed function during the endosymbiotic event that created chloroplasts provides evidence that the role is critical for the conversion of light into chemical energy. This function remains to be determined, but as is seen for cytoplasmic Get3 proteins, it is likely either involved in TA protein targeting, acts as a chaperone, or perhaps both? A completely new role is possible, and future work to identify phenotypes and interaction partners should illuminate this puzzle. The breadth of Get3-like proteins scattered across kingdoms leads to questions about where this fold first appeared and what that role may have been.

## Experimental procedures

### A reference alignment and phylogeny of the Get3/ArsA family

All proteins from UniProt version 2020_06 with an annotation as Get3/ArsA were pulled down (IPR027542, IPR016300, and IPR025723) along with all other InterPro domains identified (22, 65, 66). Get3/ArsA domains were then identified by searching against hidden Markov models for monomeric Get3/ArsA domains from solved crystal structures, split by hand for pseudodimers. The resulting domains were then searched (jackhammer, three iterations (67)) against the preliminary set of UniProt proteins to find additional monomeric representatives. Each hit that covered 90% of the best scoring query was considered complete. Pseudodimers were split midway between the end of the first domain and the start of the second domain on the parent sequence.

A reference alignment of Get3/ArsA domains was then created by clustering domains using mmseqs at 65% sequence identity (68). Clusters were then aligned using mafft, version 7.471 (25) with a seed alignment given by a structural alignment (STAMP) of Get3/ArsA proteins with solved structures (genafpair, maxiterate 1000, and retree 20) (24). A maximum-likelihood phylogeny was computed using RAxML, version 8.2.12 (26): automatic model assignment using machine learning criterion, best scoring model LG with empirical base frequencies; and rapid bootstrap search complete after 400 replicates (-I autoMRE) (69).

### Plant, photosynthetic, and αCD containing Get3/ArsA proteins

Get3/ArsA proteins with αCD domains were identified by a match to Pfam PF17886 (ArsA_HSP20) as annotated by UniProt. Organisms were defined as putatively photosynthetic if a UniProt proteome contained more than 10 proteins assigned to the Gene Ontology term GO:0015979 (photosynthesis) (70, 71). Because of uneven sequencing coverage across genomes, analysis of the presence of αCD-containing Get3 proteins in photosynthetic *versus* nonphotosynthetic organisms was carried out for proteomes annotated by UniProt as reference, nonredundant, or with a BUSCO completeness *>*75% (72).

Get3 proteins were separated into subsets to be clustered (mmseqs easycluster) into representative sequences for phylogenetic analysis with specific minimum sequence identity levels for each group: Get3 proteins from plants (85%), Get3 proteins with αCD domains from photosynthetic bacteria (70%), Get3 proteins without αCD domains from photosynthetic bacteria (65%), and all other Get3/ArsA proteins (60%). Where one of a pair of a pseudodimer was indicated as a representative, the other half was kept as well. Get3 proteins from *A. thaliana* and those with solved crystal structures were also included. The resulting sequences were then aligned using mafft (genafpair, maxiterate 1000, and retree 20) seeded by the structure-based alignment specified previously.

A maximum-likelihood phylogeny was computed using RAxML: automatic model assignment using ML criterion, best scoring model VT with fixed base frequencies; rapid bootstrap search complete after 100 replicates (-I autoMRE); N-terminal only, Get3-only, and C-terminal portion only tested as subpartitions. Nonrepresentative sequences were then added into the alignment using mafft (–add, –keeplength) and then placed onto the tree using the RAxML Evolutionary Placement Analysis (-f v) (73).

The resulting tree is *post facto* rooted using the most recent common ancestor of *E. coli* ArsA and *Mj*Get3. Plant proteins are assigned to Get3a, b/c, or d by the presence of the corresponding *A. thaliana* protein in that clade. Trees are manipulated and drawn using phyloseq (74), phytools (75), treeio (76), ggtree (77, 78), tidytree (78), and ggplot2 (79) packages in R (80) and the tidyverse (81).

### Reagents

All reagents were purchased from Sigma–Aldrich unless otherwise specified.

### Cloning

Constructs and primers used in this study are given in Table S2.

The *At*Get3d purification and crystallization construct was prepared by inserting *At*Get3dΔ1–57 into the NdeI–XhoI cut sites of pET22b(+) using standard restriction enzyme cloning methods. Based on signal/chloroplast targeting peptide prediction (40) and mafft sequence alignments (25), the first 57 amino acids were truncated from *At*Get3d.

Get3d-GFP fusion constructs were generated by isothermal assembly following standard procedures (82). Each assembled construct was in the pENTR plasmid with L1 and L2 sites that allowed for recombination of the Get3d-GFP construct into a destination vector with the UBQ10 promoter regulatory sequence and an OCS terminator sequence (pMOA pUBQ10-GW-OCS).

The *At*Get3d ATPase assay construct was prepared using standard Gibson cloning protocols (82) by inserting *At*Get3dΔ1–57 into a pET22b(+) vector containing an N-terminal His_6_ tag and human rhinovirus 3C protease cut site. The pET33b-His_6_-TEV-*Nos*Get3d ATPase assay construct was prepared using standard Gibson cloning methods (82), and pET33b-His_6_-TEV-*Sc*Get3 was prepared as described previously (32).

The Get3d TA protein capture assay constructs were prepared using standard Gibson cloning methods (82). The MBP-Sbh1 TA protein was prepared as described previously (83).

### Plant material

*N. benthamiana* seeds were germinated on Sunshine Mix 5 with perlite and vermiculite added at a ratio of 3:1:1, respectively. After seedlings germinated and the first true leaves appeared, plants were transplanted and allowed to grow for 14 days in 16:8 light:dark hour cycle.

### *Agrobacteria* transformation and tobacco infiltration of Get3d-GFP variants

For transient expression, plasmids pUBQ10::Get3d-GFP and pUBQ10::Get3dΔTP-GFP were introduced into the *Agrobacterium* strain GV3101 by triparental mating. *Agrobacteria* strains were grown in 2xYT media with gentamycin (30 μg/ml), rifampicin (50 μg/ml), and spectinomycin (100 μg/ml) and adjusted to an absorbance of 0.1 in 10 mM MgCl_2_ and 150 μM acetosyringone. About 1 ml of each *Agrobacterium* sample was infiltrated into each leaf using a 1 ml syringe. Three to four leaves were infiltrated per construct.

### Confocal microscopy

After 48 h, tobacco leaf samples were imaged on an upright Zeiss 780 Confocal Laser Scanning Microscope. The 488 nm laser line was used to excite both GFP and induce chlorophyll autofluorescence. Standard excitation and emission windows were used for GFP and chlorophyll b. Microscopy images were processed in ImageJ (84, 85).

### *At*Get3dΔ1–57 expression, purification, and crystallization

*At*Get3dΔ1–57 was expressed in *E. coli* BL21(DE3) Star. Briefly, 100 ml of LB with ampicillin was inoculated with overnight culture (1%) and grown at 37 °C. At an absorbance of ∼1.0 at 600 nm, protein expression was induced by adding 0.4 mM IPTG, and the culture was incubated overnight at 16 °C. After overnight induction, cells were harvested by centrifugation, resuspended in lysis buffer (25 mM Tris–HCl [pH 7.5], 500 mM NaCl, 30 mM imidazole, 2 mM β-mercaptoethanol, 0.1% Triton X-100, 2 mM MgCl_2_, and 10% glycerol), and sonicated for 5 min with 5 s on/off pulse and 45% amplitude. Cell debris was pelleted by centrifugation at 13,000*g* for 30 min, and the clarified lysate was passed over a pre-equilibrated nickel–nitrilotriacetic acid column (Qiagen). The column was washed with 10 column volumes wash buffer (25 mM Tris–HCl [pH 7.5], 500 mM NaCl, 30 mM imidazole, 2 mM β-mercaptoethanol, 2 mM MgCl_2_, and 10% glycerol) and eluted by raising to 250 mM imidazole. Elution fractions were pooled and concentrated using Amicon 30k molecular weight cutoff (MWCO) concentrator (Sigma–Aldrich). The concentrated protein was further purified through a pre-equilibrated (25 mM Tris–HCl [pH 7.5], 150 mM NaCl, 2 mM DTT, 1 mM MgCl_2_, and 10% glycerol) Superdex 200 10/300 GL (Cytiva) size-exclusion column.

Fractions were pooled and further concentrated to 10 mg/ml for crystallization trials by sitting drop vapour diffusion. Crystals were grown at room temperature by mixing equal volume of protein solution containing 2 mM ADP/adenylyl imidodiphosphate with reservoir solution containing 50 mM sodium cacodylate (pH 5.47), 50 mM lithium sulfate, and 30% PEG-4000. Crystals were cryoprotected in the mother liquid supplemented with 30% glycerol before flash-freezing in liquid nitrogen.

### Data collection, structure solution, and refinement

*At*Get3d data collection was done at European Synchrotron Radiation Facility beamline BM-14 at 100 K and 0.97625 Å. The data were integrated with XDS and scaled with Aimless (CCP4 suite) in space group P 1 2_1_ 1 to a resolution of 2.0 Å (86, 87, 88). Data collection and refinement statistics are listed in Table S1.

The *At*Get3d structure was determined by molecular replacement with PHASER using the Get3d homodimer from *Nostoc* sp. PCC 7120 (PDB ID: 3IGF) as the search model (43, 89). Several rounds of model building and refinement were carried out with phenix.refine, CCP4/Refmac, and COOT (90, 91, 92, 93, 94). A single molecule of an *At*Get3d dimer was found in the asymmetric unit. Side-chain density was generally weak in the α-helical subdomains, and density was missing for residues 250 to 260, 330 to 331, and 378 to 382 in monomer A and residues 252 to 261 and 380 to 384 in monomer B. For *At*Get3d, residue numbering includes the chloroplast targeting peptide.

The *Nostoc* sp. PCC 7120 Get3d structure (PDB ID: 3IGF) was refined using phenix.refine, and manual building was performed in COOT (90, 91, 92). Residues 160 to 182 from monomer A and 88 to 89 and 167 to 182 from monomer B were added in this study. Residue 195 from monomer A was removed in our refinement because of poor density. Density was missing for residues 183 to 195 in monomer A and 183 to 192 in monomer B. *Nos*Get3d data collection information is available at PDB ID: 3IGF (43). Data collection and refinement statistics are listed in Table S1.

Structural figures were generated using PyMOL (https://pymol.org/) (95).

### Native mass spectrometry

His_8_-SUMO-GSx2-*At*Get3dΔ1–57 was expressed in NiCo21(DE3) *E. coli* in 2xYT. At an absorbance of ∼0.7 at 600 nm, protein expression was induced by adding 0.4 mM IPTG, and the culture was incubated for 4 h at 37 °C. Cells were harvested and lysed in 50 mM Tris–HCl (pH 7.5), 300 mM NaCl, 20 mM imidazole, 10 mM β-mercaptoethanol, 1 mM PMSF, and 1 mM benzamidine in a Microfluidizer (Microfluidic). After cell debris was pelleted, the clarified lysate was passed over 500 μl per 1 l culture of pre-equilibrated nickel–nitrilotriacetic acid resin, washed with 100 column volume wash buffer (50 mM Tris–HCl [pH 7.5], 300 mM NaCl, 20 mM imidazole, 10 mM β-mercaptoethanol, 1 mM PMSF, and 1 mM benzamidine), and eluted with elution buffer (20 mM Tris–HCl [pH 7.5], 150 mM NaCl, 300 mM imidazole, and 10 mM β-mercaptoethanol). The nickel affinity chromatography elution fractions were pooled and dialyzed with 0.02 mg/ml Ulp1 against Get3 buffer (50 mM Hepes [pH 7.5], 150 mM KOAc, 5 mM Mg(OAc)_2_, and 10 mM β-mercaptoethanol) in 30 kDa MWCO Snakeskin Dialysis Tubing (Thermo Fisher Scientific) overnight at 4 °C. Protein was concentrated in an Amicon 30k MWCO concentrator (Sigma–Aldrich) and then purified by size-exclusion chromatography using a Superdex 200 10/300 GL size-exclusion column (Cytiva) in Get3 buffer. Fractions were pooled and concentrated to 50 μM for mass spectrometry analysis.

Samples were prepared as previously described (96). In brief, protein samples were buffer exchanged into MS buffer (200 mM ammonium acetate [pH 7.4]) with a centrifugal desalting column (Micro Bio-Spin 6 Columns; Bio-Rad). Mass spectra were collected on a Q Exactive UHMR Hybrid Quadrupole-Orbitrap Mass Spectrometer (Thermo Fisher Scientific). Samples were loaded into a gold-coated borosilicate glass capillary prepared in-house and were introduced into the mass spectrometer *via* nano electrospray ionization. The mass spectrometry conditions were set as follows: in-source trapping off, high *m/z* setting for ion transfer target range, injection flatapole DC 5 V, inter flatapole lens 4 V, bent flatapole DC, and transfer multipole DC were both 0 V. The higher-energy collisional dissociation (HCD) events were set as follows: HCD time 3 ms, purge time 20 ms, HCD field gradient 200 V, and trapping gas pressure was set to 5. Native mass spectra were collected with an *m/z* range from 500 to 15,000, resolution at 12,500, one microscan, spray voltage of 1.6 kV, capillary temperature of 100 °C, and a maximum inject time of 500 ms. The CE varied from 50 V to 200 V. The raw spectra were processed and deconvoluted using UniDec (97).

### ATPase activity assay

His_6_-3C-*At*Get3dΔ1–57, His_6_-TEV-*Nos*Get3d, or His_6_-TEV-*Sc*Get3 (yeast Get3) were expressed and purified as described previously with 1 mM IPTG. Subsequent experiments were carried out in the same fashion for each construct. The nickel affinity chromatography elution fractions were run on 15% SDS-PAGE, pooled fractions were concentrated in an Amicon 30k MWCO concentrator (Sigma–Aldrich), and protein concentration was determined using a NanoDrop spectrophotometer (Thermo Fisher Scientific).

ATPase activity was determined at 37 °C using the EnzChek Phosphate Assay Kit (Thermo Fisher Scientific), a microplate spectrophotometric assay that couples inorganic phosphate production to the enzymatic conversion of 2-amino-6-mercapto-7-methyl-purine riboside to ribose 1-phosphate and 2-amino-6-mercapto-7-methylpurine by purine nucleoside phosphorylase (PNP) (98). About 100 μl reactions were carried out with Get3 (either 15 μM *At*Get3d, 15 μM *Nos*Get3d, or 2.5 μM *Sc*Get3), 0.2 mM 2-amino-6-mercapto-7-methyl-purine riboside, 1 U/l PNP, 5 mM MgCl_2_, and 0 μM, 37.25 μM, 62.5 μM, 125 μM, 250 μM, 500 μM, 1 mM, or 2 mM ATP in Get3 buffer (described previously). Reactions were initiated with the addition of Get3d or Get3, and the absorbance was measured at 360 nm every 20 s for a total of 6.67 min using a Tecan Infinite M Nano+ plate reader in 96-well plates (Corning Costar 96-Well Plate; Thermo Fisher Scientific). The method was programmed using Magellan, version 7.2 software (Tecan). Measurements were taken in triplicate at each concentration.

As a control, reactions were performed as described previously with 0 μM, 1.56 μM, 3.13 μM, 6.25 μM, 12.5 μM, 25 μM, 50 μM, or 100 μM P_i_ without Get3 or ATP. Reactions were initiated with the addition of P_i_. Maximal absorbance at 5 min was plotted against concentration P_i_ (μM), fit with a linear trendline (A_360_ = 0.0020198 × [P_i_ (μM)] + 0.0043, *R*^2^ = 0.9937), and utilized to determine A_360_/nmol P_i_.

The resultant data were corrected for background absorbance (A_360_ at 0 μM ATP), analyzed using ICEKAT (https://icekat.herokuapp.com/icekat) (99), and plotted with the Altair Python package (100).

### Coexpression and pulldown of TA protein by *At*Get3d and *Nos*Get3d with and without αCD

pET33b-His_6_-TEV-tagged *At*Get3dΔ1–57, *At*Get3dΔ1–57ΔαCD, *Nos*Get3d, or *Nos*Get3dΔαCD were expressed in *E. coli* BL21(DE3) Star with or without the pACYCDuet-MBP-tagged yeast TA protein Sbh1 (32, 83) and purified as described previously with the following changes: 0.2 mM IPTG was used, and cultures were incubated overnight at 18 °C. Subsequent experiments were carried out in the same fashion for each construct. The nickel affinity chromatography elution fractions were run on 15% SDS-PAGE, normalizing for the amount of Get3d in each sample by integrating the Get3d band intensity in ImageJ (84, 85). Identity of the bands was confirmed by Western blot with the samples run on a 15% SDS-PAGE and then transferred to 0.45 μm nitrocellulose membranes using the Transblot Turbo System (Bio-Rad). The membranes were blocked using 5% dry milk in TTBS (20 mM Tris–HCl [pH 7.5], 150 mM NaCl, 0.1% Tween-20) at room temperature for 1 h followed by incubation with either an anti-His_5_ antibody for Get3d (from mouse) (Sigma–Aldrich; catalog no.: SAB1305538) or an anti-MBP antibody for the TA protein (from mouse) (New England Biolabs; catalog no.: E8032) in 5% dry milk in TTBS overnight at 4 °C. Bands were visualized after rinsing with TTBS (3 × 5 min) and then incubating with anti-mouse antibody (from rabbit) conjugated to Alkaline Phosphatase (Rockland Immunochemicals; catalog no.: 610-4512) (in 5% dry milk in TTBS) for 3 h at room temperature. The membranes were rinsed with TTBS (3 × 5 min) and AP Developing Buffer (100 mM Tris–HCl [pH 9.5], 100 mM NaCl, and 5 mM MgCl_2_, 1 × 5 min), developed using AP Substrate (0.33 mg/ml NBT and 0.165 mg/ml BCIP) in AP Developing Buffer, and imaged using a ChemiDoc MP Imaging System (Bio-Rad).

## Data availability

Data and materials are available from the corresponding author(s) upon request.

## Supporting information

This article contains supporting information.

## Conflict of interest

The authors declare that they have no conflicts of interest with the contents of this article.
