# Supplementary material for: Structures of Get3d reveal a distinct architecture associated with the emergence of photosynthesis
Source: J Biol Chem. 2023 Apr 24;299(6):104752. doi: 10.1016/j.jbc.2023.104752 (PMC10248533; doi:10.1016/j.jbc.2023.104752)
Supplement: Supporting information [file mmc1.pdf]

Supporting Information

Structures of Get3d reveal a distinct architecture associated with the emergence of photosynthesis

Alexandra N. Barlow, M. S. Manu, Shyam M. Saladi, Paul T. Tarr, Yashpal Yadav, Aye M. M. Thinn, Yun Zhu, Arthur D. Laganowsky, William M. Clemons Jr., and Sureshkumar Ramasamy

Fig. S1 A detailed phylogeny of Get3 homologs.

Fig. S2 A detailed phylogenetic analysis of Get3 homologs.

Fig. S3 A detailed cladogram of Get3 homologs in plants.

Fig. S4 Details of the *At*Get3d structure.

Table S1 Data collection and refinement statistics.

Fig. S5 View of the missing A-loop and CXXC motif.

Fig. S6 Possible lipids in the hydrophobic chamber of Get3d.

Fig. S7 New refinement of *Nos*Get3d.

Fig. S8 Sequence alignment of selected αCDs.

Fig. S9 Interactions at the αCD interface.

Fig. S10 The hydrophobic chamber of Get3d.

Table S2 Constructs and primers utilized in this study.

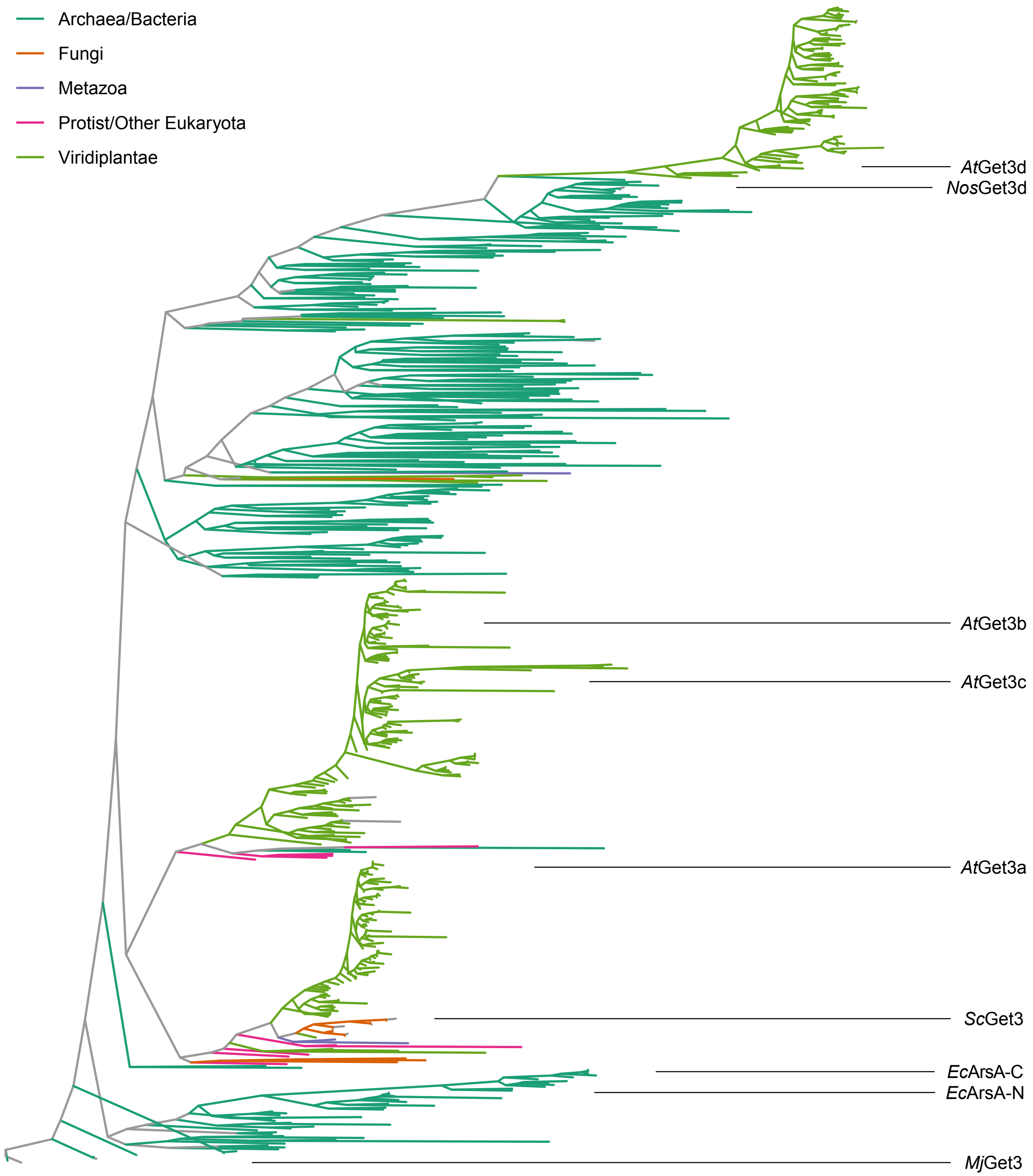

Fig. S1 A detailed phylogeny of Get3 homologs. Get3 proteins from notable species are labeled to the *right* with species abbreviations as in Fig. 1. Branches are colored by taxonomic grouping: Archaea/Bacteria (*blue-green*), Fungi (*orange*), Metazoa (*purple*), protists/other Eukaryota (*pink*), Viridiplantae (*green*). Inner branches are colored where all descendants are of a single taxonomic grouping.

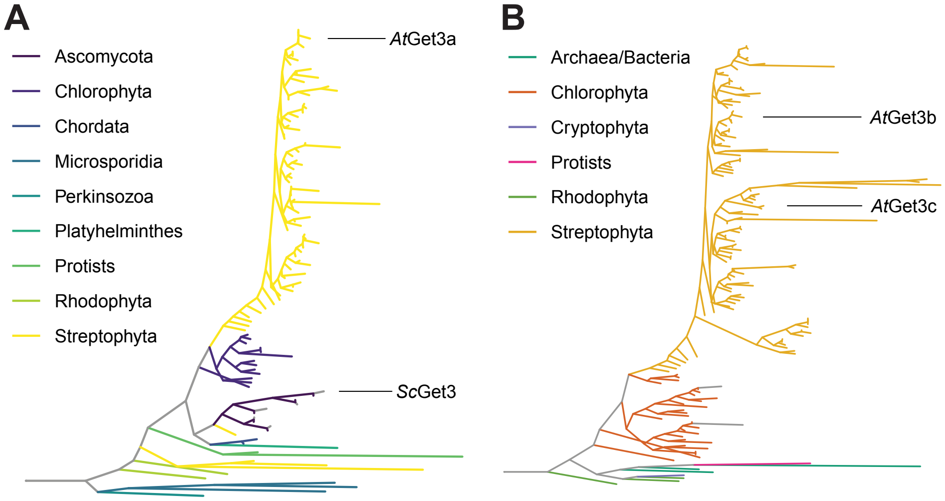

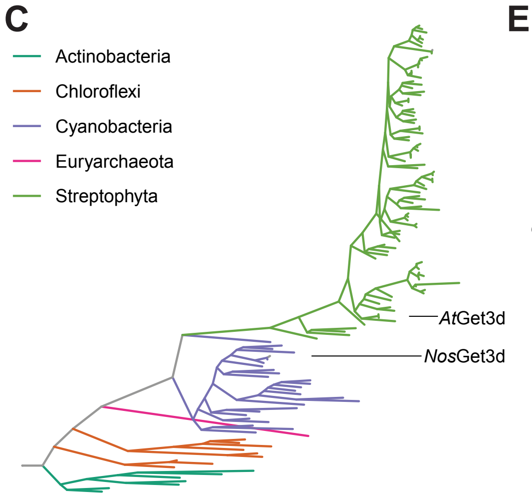

E

	Number of Get3/ArsA Proteins	
	without αCD	with αCD
Chlorobium chlorochromatii	0	5
Roseiflexus sp.	0	2
Gloeobacter violaceus	0	2
Nostoc punctiforme	1	2
Trichodesmium erythraeum	1	2
Prochlorococcus marinus	0	0
Synechococcus sp.	0	2
Gemmatimonas phototrophica	1	0
Acidithiobacillus ferrooxidans	1	0
Rhodopseudomonas palustris	0	0
Roseateles depolymerans	0	0
Allochromatium warmingii	0	0

D

Phylum	Family	Number of Genomes with a Get3/ArsA without αCD	with αCD	
Chlorobi	*Chlorobiaceae*	0	2	Green sulfur
Chloroflexi	*Chloroflexia*	0	1	Green non-sulfur
Cyanobacteria	*Chroococcidiopsidales*	0	2	
	Gloeobacteria	0	2	
	Gloeoemargaritales	0	1	
	Nostocales	0	33	
	Oscillatoriophycideae	0	37	
	Pleurocapsales	0	4	
	Synechococcales	0	24	
Proteobacteria	*Acidithiobacillia*	1	0	
	Alphaproteobacteria	8	1	Purple non-sulfur
	Betaproteobacteria	13	0	
	Gammaproteobacteria	7	0	Purple sulfur
Gemmatimonadetes	*Gemmatimonadales*	1	0	

Fig. S2 A detailed phylogenetic analysis of Get3 homologs. Get3 proteins from notable species are labeled to the *right* with species abbreviations as in Fig. 1. Phylogenetic tree of (A) Get3a, (B) Get3b/c, and (C) Get3d paralogs, colored by taxonomy. Phyla are colored as follows: (A) Ascomycota (*dark purple*), Chlorophyta (*purple*), Chordata (*blue*), Microsporidia (*dark blue-green*), Perkinsozoa (*light blue-green*), Platyhelminthes (*dark green*), protists (*green*), Rhodophyta (*light green*), Streptophyta (*yellow*); (B) Archaea/Bacteria (*dark green*), Chlorophyta (*dark orange*), Cryptophyta (*purple*), protists (*pink*), Rhodophyta (*green*), Streptophyta (*orange*); (C) Actinobacteria (*blue-green*), Chloroflexi (*orange*), Cyanobacteria (*light purple*), Euryarchaeota (*pink*), Streptophyta (*green*). Inner branches are colored where all descendants are of a single taxonomic grouping. (D) Number of reference/complete genomes that contain Get3/ArsA homologs with and without the characteristic αCD in various phyla. (E) Number of Get3/ArsA homologs with and without the αCD encoded by representative species from the phyla in (D).

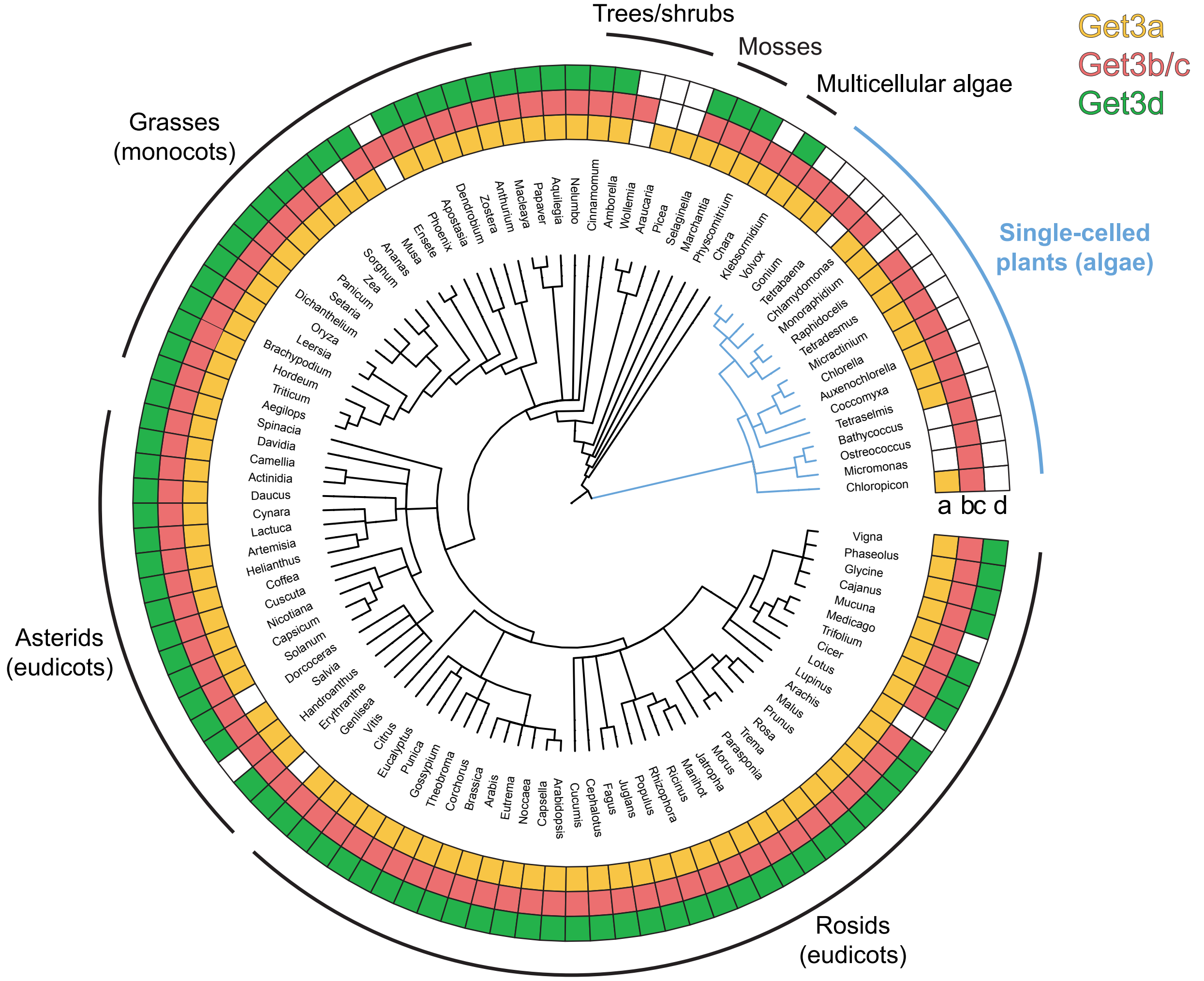

Fig. S3 A detailed cladogram of Get3 homologs in plants. The presence of Get3 homologs is shown as in Fig. 1C. Genera are labeled on the interior of the circle. Selected taxa/clades are labeled on the exterior of the circle.

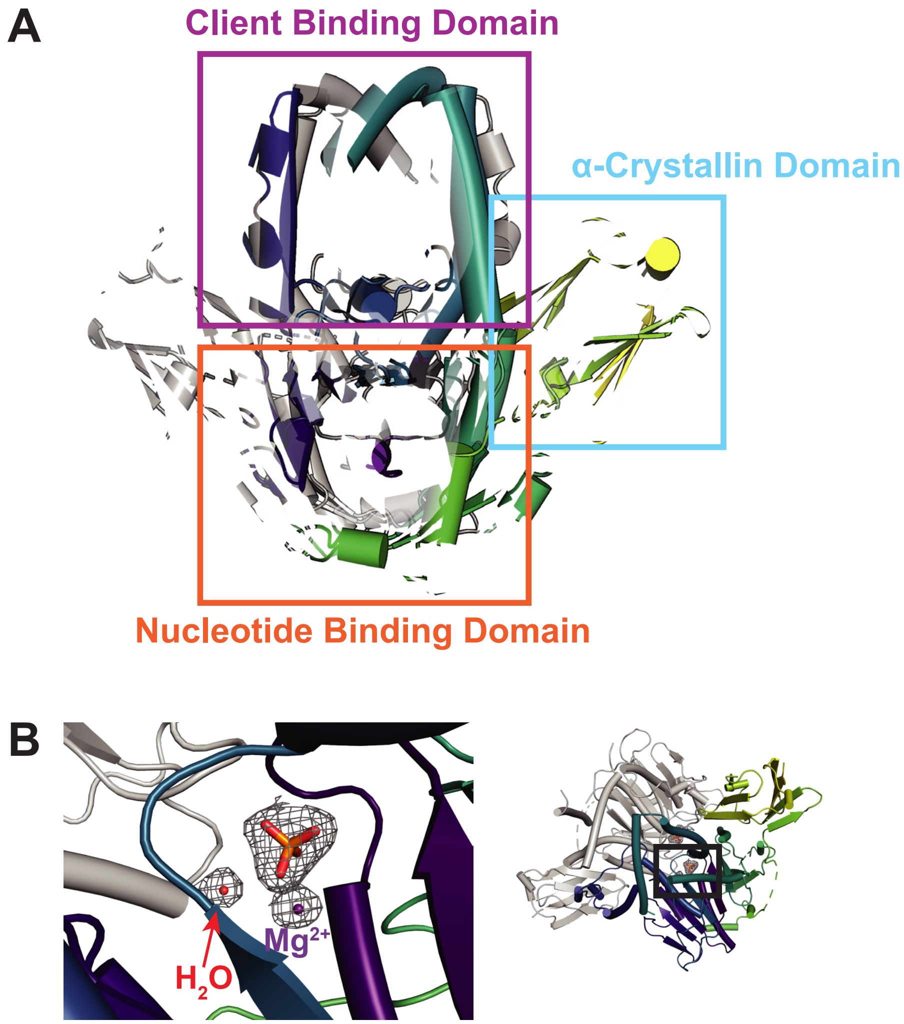

Fig. S4 Details of the *At*Get3d structure. (A) View from the *front* of *At*Get3d. The client binding, nucleotide binding, and α-crystallin domains are indicated. (B) Active site of *At*Get3d showing $2F_O$-F_C electron density at 1σ (*grey mesh*) for water (*red sphere*), Mg^{2+} (*purple sphere*), and phosphate (*sticks*). The region highlighted is shown on the *right* in the full structure. *At*Get3d colored as in Fig. 3.

Table S1. Data collection and refinement statistics.

	*At*Get3d<br>PDB ID: 8ELF	*Nos*Get3d<br>PDB ID: 8EGK†	*Nos*Get3d<br>PDB ID: 3IGF††
**Data Collection**			
Space Group	P 1 2_1 1		C 1 2 1
Cell dimensions			
a, b, c (Å)	59.25, 67.05, 99.40		124.17, 55.55, 122.36
α, β, γ (°)	90.00, 97.81, 90.00		90.00, 98.87, 90.00
Resolution (Å)	58.70 - 2.00 (2.05 - 2.00) *		30.00 - 2.00 (2.07 - 2.00)
R_{merge}	0.061 (1.863)		0.063 (0.288)
I/σ	10.3 (0.6)		18.8 (3.90)
Completeness (%)	99.42 (99.25)		95.22 (91.54) **
Redundancy	4.2 (4.2)		3.3 (3.1)
**Refinement**			
Resolution (Å)	32.83 - 2.00	27.52 - 1.98	19.96 – 2.00
No. reflections	52,063	54,162	53,378 **
R_{work} / R_{free}	0.215 / 0.258	0.189 / 0.221	0.189 / 0.233
No. non-hydrogen atoms	6112	5873	5680
Protein	5955	5528	5207
Ligand	116	6	0
Solvent	100	339	473
Avg. B factors	59.52	42.26	36.76
Protein	59.63	42.55	36.46
Ligand	75.78	41.34	
Solvent	43.66	37.47	40.08
R.m.s. deviations			
Bond length (Å)	0.004	0.007	0.017
Bond angles (°)	0.62	0.76	1.71
**Validation**			
MolProbity Score	1.35	1.15	2.49
Rotamer outliers (%)	0.92	0.33	8.13
Cβ outliers	0	0	1
Ramachandran outliers (%)	0.00	0.00	0.15
Ramachandran favored (%)	97.47	97.86	96.97
Ramachandran allowed (%)	2.53	2.42	2.88
Rama-Z	-1.21 ± 0.27	-0.90 ± 0.30	-0.97 ± 0.30

*At*Get3d dataset was collected from a single crystal and structure was determined by molecular replacement using PDB ID: 3IGF as the search model. †*Nos*Get3d re-refinement and validation statistics from this study. ††*Nos*Get3d data collection, refinement, and validation statistics from PDB ID: 3IGF. *Values in parenthesis are for the highest resolution shell. **Values determined using Phenix.

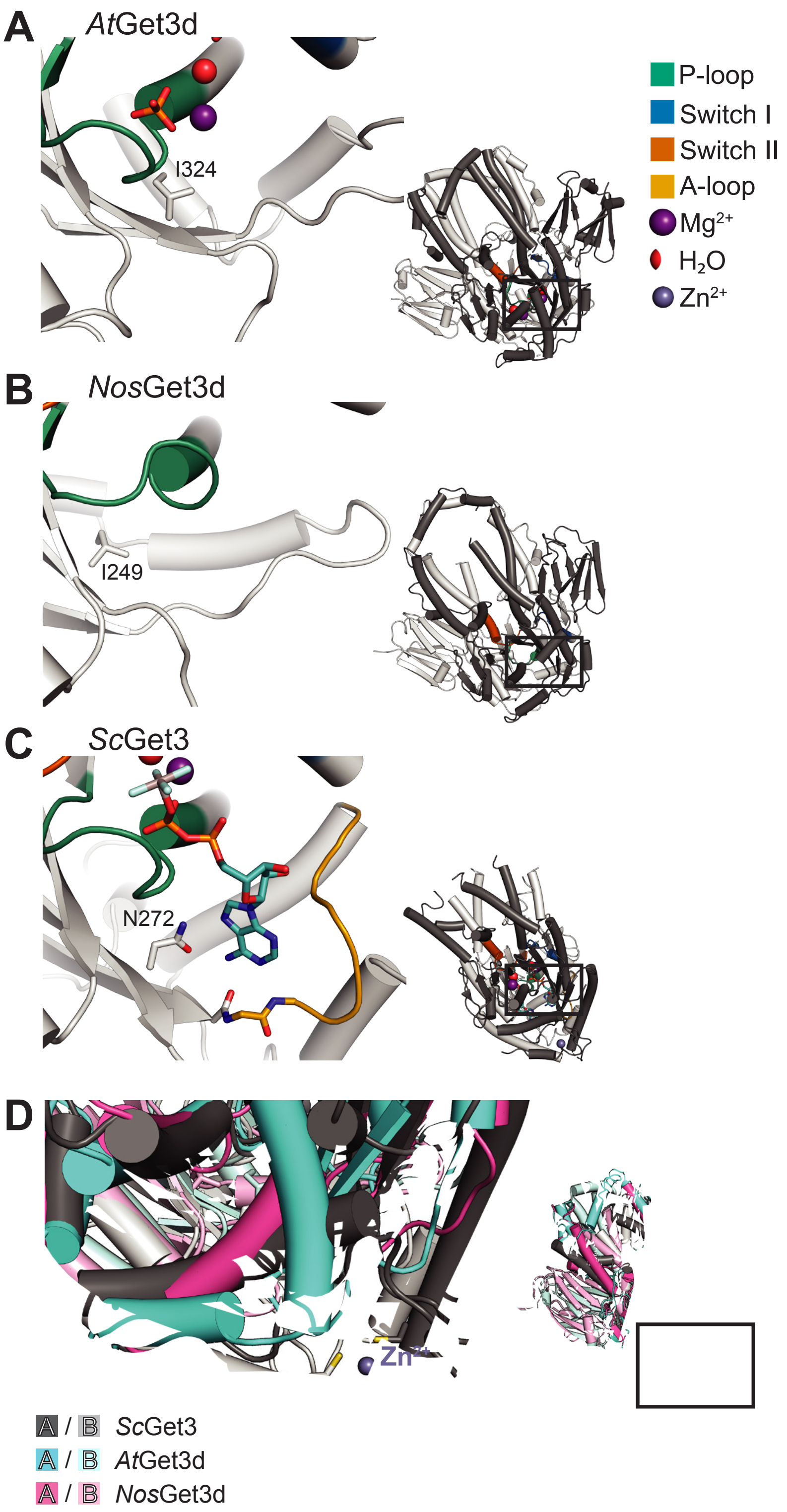

Fig. S5 View of the missing A-loop and CXXC motif. Structure of the active site of a monomer of (A) *At*Get3d, (B) *Nos*Get3d, and (C) the closed conformation of yeast Get3 (*Sc*Get3, PDB ID: 2WOJ) showing the A-loop and asparagine missing in Get3d. Colored as in Fig. 5. (D) Structure of the CXXC motif of the closed conformation of *Sc*Get3 (PDB ID: 2WOJ, *dark and light grey*), with cysteine residues (*sticks*) coordinating a Zn^{2+} ion (*grey sphere*). The structures of *At*Get3d (*dark and light aquamarine*) and *Nos*Get3d (*dark and light pink*) are aligned. The region highlighted is shown on the *right* in the full alignment.

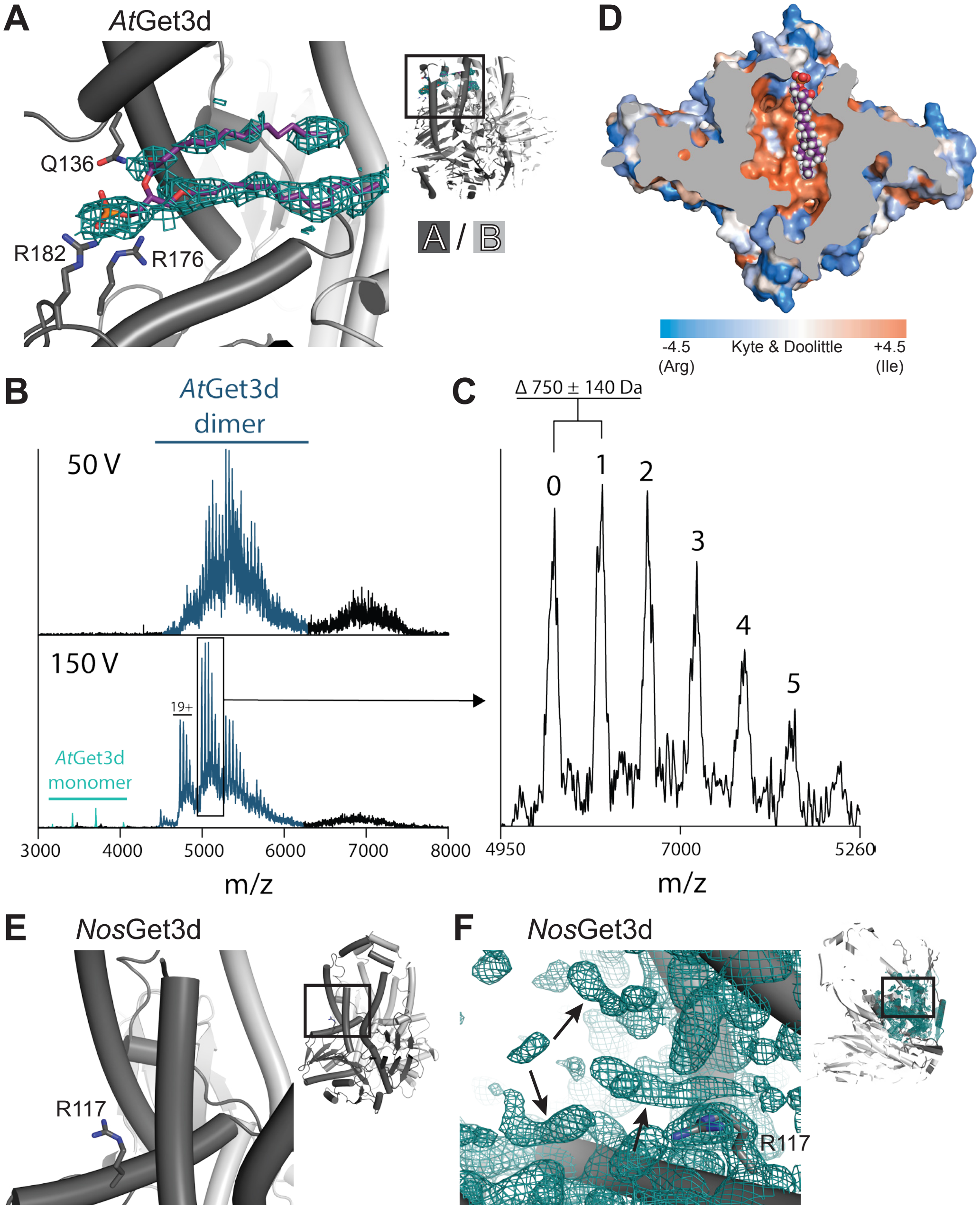

Fig. S6 Possible lipids in the hydrophobic chamber of Get3d. (A) 2Fo-Fc electron density (*teal mesh*) in the bottom of the hydrophobic chamber of *At*Get3d near the refined phosphatidic acid (*sticks* with two fourteen carbon saturated acyl chains with *purple* carbons) contoured at 1σ. Monomer A and B are shown in *grey and light grey*, respectively. Side chains for residues that coordinate the putative phosphate head group are shown as *sticks*. The region highlighted is shown on the *right* in the full structure. (B) Native mass spectra of *At*Get3d acquired under different activation energies. The theoretical and measured dimer masses are 88,878 Da and 89,944 Da, respectively. (C) Deconvolution of the mass spectrum shown in (B) acquired at CE of 150V. Multiple adducts are bound to the protein with a mass of 750±140 Da. (D) Slice through view of the *At*Get3d accessible surface in the bottom of the hydrophobic chamber colored by hydrophobicity using the Kyte and Doolittle scale with the phosphatidic acid shown as *spheres*. (E) Structure of *Nos*Get3d showing the conserved arginine in *sticks*. The region highlighted is shown on the *right* in the full structure. (F) 2Fo-Fc electron density map (*teal mesh*) in the bottom of the hydrophobic chamber of *Nos*Get3d contoured at 1σ. Arrows point to densities that were unidentified in our refinement. (E) and (F) colored as in (A).

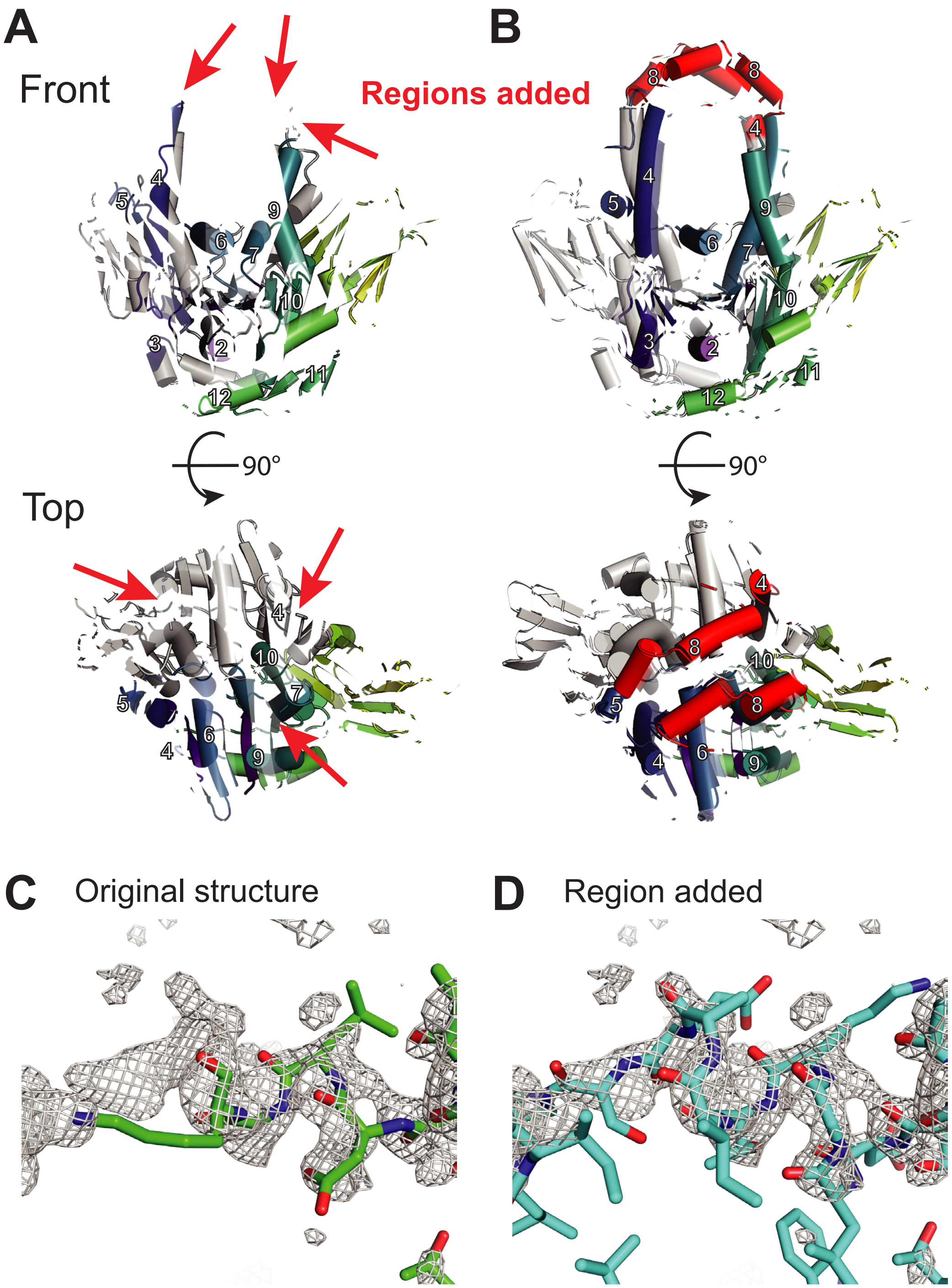

Fig. S7 New refinement of *Nos*Get3d. (A) Original deposited structure of *Nos*Get3d (PDB ID: 3IGF) and (B) structure after refinement here. The *front* and *top* views are shown. Highlighted *red* regions were added in this refinement. Color and TMD numbering as in Fig. 3. (C) 2Fo-Fc electron density map (*light grey mesh*) and model of the original deposited structure of *Nos*Get3d (*green sticks*) and (D) the structure after refinement (*blue sticks*), showing the improved fit and residues added. 2Fo-Fc electron density map contoured at 1σ.

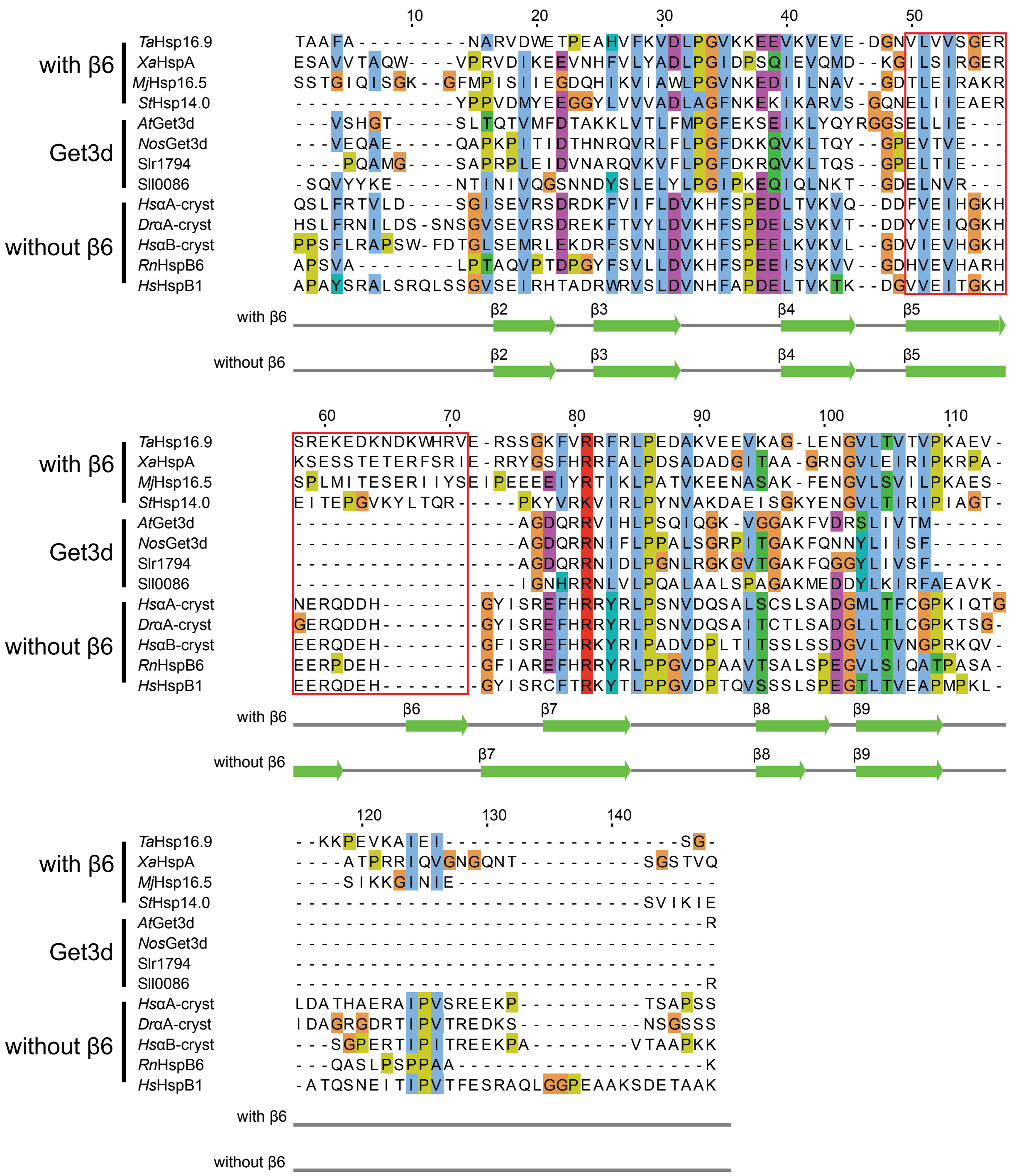

Fig. S8 Sequence alignment of selected αCDs. Residues colored as in Fig. 1B. Predicted secondary structure is shown below the sequences for αCD with and without the loop containing β6. Sequences are classified as either αCD with or without the loop containing β6 or αCD of Get3d. Discussed regions are boxed in red. Species key: *Ta*, *Triticum aestivum*; *Xa*, *Xanthomonas axonopodis*; *St*, *Sulfurisphaera tokodaii*; *Dr*, *Danio rerio*; *Rn*, *Rattus norvegicus*. Coloring and other species as in Fig. 1B.

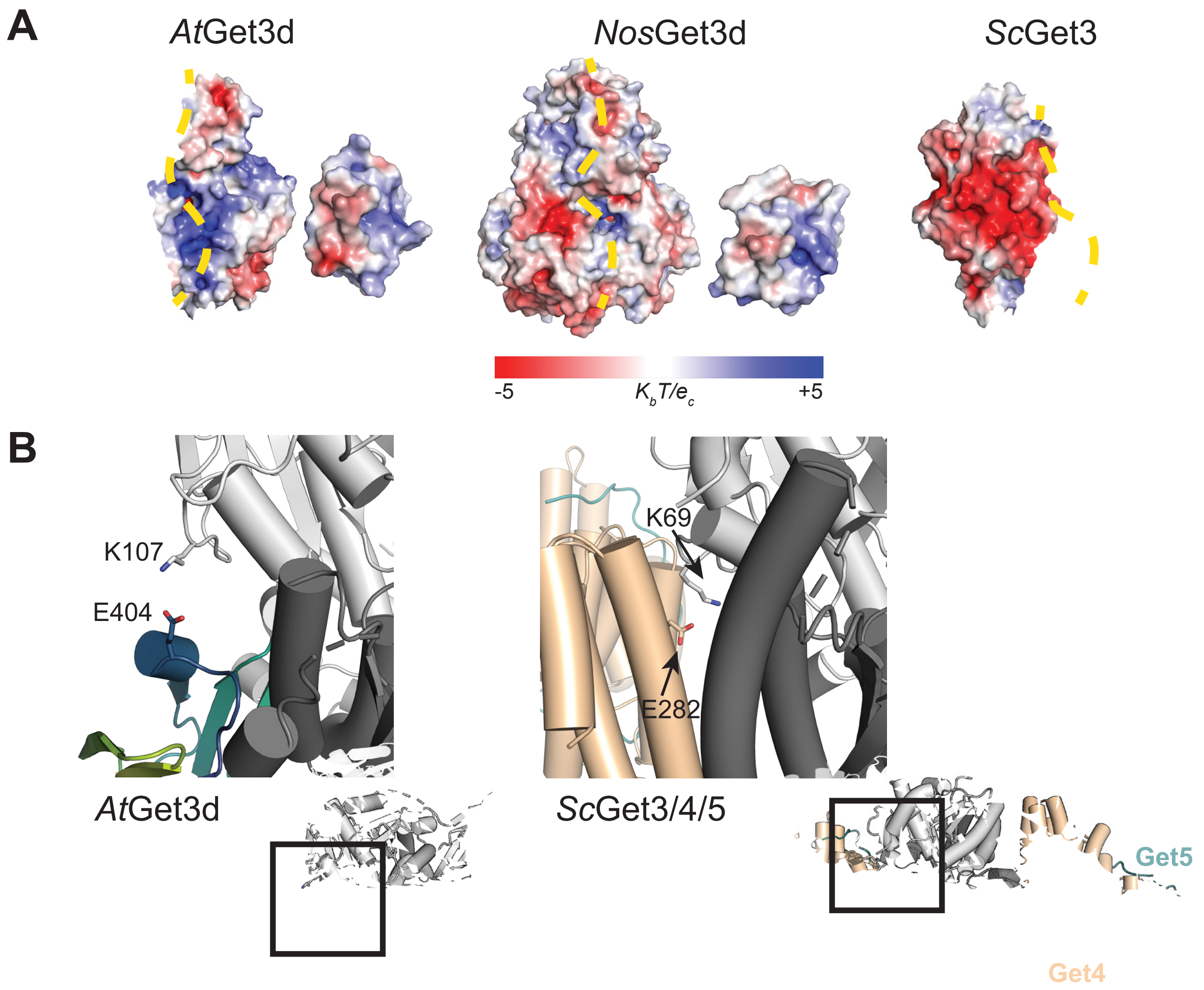

Fig. S9 Interactions at the αCD interface. (A) Electrostatic potential showing the interactions between the αCD of *At*Get3d (*left*), *Nos*Get3d (*middle*), and yeast Get3 (*Sc*Get3) with Get4/5 (*right*). Orientation of the Get3 domain (*left*) as in Fig. 4D with αCD reflected (*right*) to show the surface that interacts with the Get3 domain. The electrostatic surface was calculated using PDB2PQR and Adaptive Poisson-Boltzmann Solver (APBS) in Pymol, scale shown. The *dotted yellow line* traces the interface between the two monomers of the Get3 domain. (B) Structure of *At*Get3d (*left*) showing a salt bridge between the Get3 domain and the αCD of the opposite monomer. Colored as in Fig. 4A. Structure of yeast Get3 (*right*, *dark and light grey*) in complex with Get4/5 (*wheat* and *teal*, respectively) (*Sc*Get3/4/5) showing a salt bridge formed between Get3 and Get4. Discussed residues are shown as *sticks*. For each, the region highlighted is shown below in the full structure.

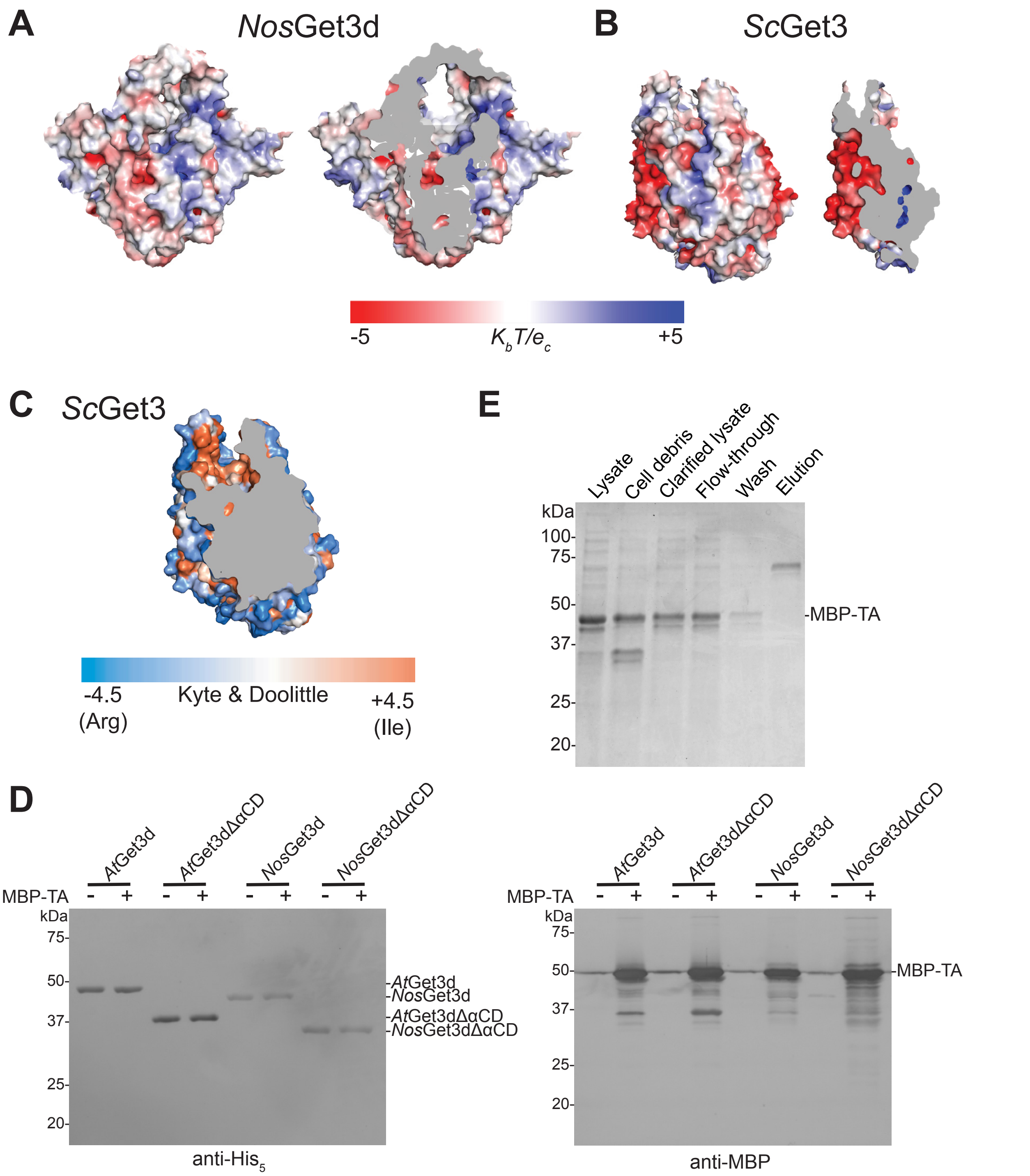

Fig. S10 The hydrophobic chamber of Get3d. Full (*left*) and slice view (*right*) surface electrostatic potential of (A) *Nos*Get3d and (B) the closed conformation of yeast Get3 (*Sc*Get3, PDB ID: 4XTR) as in Fig. 6A. (C) Slice view of the surface hydrophobicity of the closed conformation of yeast Get3 (*Sc*Get3, PDB ID: 4XTR) as in Fig. 6B. (D) Anti-His_5 (*left*) and anti-MBP (*right*) Western blots of samples from Fig. 6D. Get3d is identified using an anti-His_5 antibody, and TA protein is identified using an anti-MBP antibody. (E) Coomassie stained SDS-PAGE of MBP-TA without Get3d treated as in Fig. 6D.

Table S2 Constructs and primers utilized in this study.

Construct	Primer	Notes
pET22b-*At*Get3dΔ1-57-His$_6$	F:GCTTCATATGACCAAATTCGTCACCTTTCTCGG	NdeI/XhoI
	R:CGGAAGCTTCCGCATTGTGACGATGAGAC	
pUBQ10-gGet3d-EGFP	F:TTATAATGCCAACTTTGTACAAAAAAGCAGAAAAAAATGGTGTCTTTGGTCAATTC	*Arabidopsis thaliana* genomic DNA amplification
	R:AGATCCAGCAGATCCCCGCATTGTGACGATGAG	
	F:AAAAAAATGGTGTCTTTGGTCAATTCTTCT	
	R:CCGCATTGTGACGATGAGACT	
pUBQ10-gGet3dΔ1-34-EGFP	F:TTATAATGCCAACTTTGTACAAAAAAGCAGAAAAAAATGGTGGCAGCTTATG	*Arabidopsis thalian*a genomic DNA amplification
	R:AGATCCAGCAGATCCCCGCATTGTGACGATGAG	
	F:AAAAAAATGGTGGCAGCTTATGTGGCGGCTAC	
	R:CCGCATTGTGACGATGAGACT	
pUBQ10-gGet3d-EGFP & pUBQ10-gGet3dΔ1-34-EGFP	F:CAGCTTTCTTGTACAAAGTTG	pENTR amplification
	R:CTGCTTTTTTGTACAAAGTTG	
	F:ATCGTCACAATGCGGGGATCTGCTGGATCTGCTGCTGGATCTGGAGAATTTATGGTGAGCAAGGGCGAG	GFP amplification
	R:TTATAATGCCAACTTTGTACAAGAAAGCTGTTACTTGTACAGCTCGTCC	
	F:AAAAATGGTGGCAGCTTATGTGGCGGCTAC	Sequencing
	R:CATAAGCTGCCACCATTTTTTTCTGCTTTTTTGTACAAAGTTG	
pET22b-His$_6$-3C-*At*Get3dΔ1-57	Vector F:GTCACAATGCGGTAGTCTCGAGCACCACCAC	Gibson cloning
	Vector R:GACGAATTTGGTACTTCCGCTGCCTGGTC	
	Insert F:ACCAAATTCGTCACCTTTCTCG	
	Insert R:CCGCATTGTGACGATGAGAC	
pET33b-His$_6$-TEV-*At*Get3dΔ1-57	F:CAGAGCGTCGACACCAAATTCGTCAC	*At*Get3dΔ1-57 amplification for Gibson cloning
	R:TCCCATATGCTACCGCATTGTGACG	
pET33b-His$_6$-TEV-*At*Get3dΔ1-57,377-485	F:CAGAGCGTCGACACCAAATTCGTCAC	*At*Get3dΔ1-57,377-485 amplification for Gibson cloning
	R:TTCCCATATGCTAAGTTTCAGAGAGAAGTTC	
pET33b-His$_6$-TEV-*Nos*Get3d	F:CAGAGCGTCGACGCCCTGATATTGAC	*Nos*Get3d amplification for Gibson cloning
	R:TCCCATATGCTACTCGAGGAAAGAAATGATC	
pET33b-His$_6$-TEV-*Nos*Get3dΔ292-366	F:CAGAGCGTCGACGCCCTGATATTGAC	*Nos*Get3dΔ292-366 amplification for Gibson cloning
	R:TCCCATATGCTACGCTTGTTCGGCTTG	
pET33b-His$_6$-TEV Get3d constructs	F:TAGCATATGGGAATTCGAAGCTTGCGG	pET33b-His$_6$-TEV vector amplification for Gibson cloning
	R:GTCGACGCTCTGGAAGTACAGGTTTTC	
pET33b-His$_6$-TEV-*Sc*Get3		See Suloway 2009
pACYCDuet-MBP-*Sc*Sbh1(52-82)		See Lin 2021

"F" corresponds to forward primer. "R" corresponds to reverse primer. All primers 5' to 3'.
